# Trans-generational immune priming against American Foulbrood does not affect the performance of honeybee colonies

**DOI:** 10.3389/fvets.2023.1129701

**Published:** 2023-02-27

**Authors:** Matti Leponiemi, Helena Wirta, Dalial Freitak

**Affiliations:** ^1^Institute of Biology, University of Graz, Graz, Austria; ^2^Department of Agricultural Sciences, University of Helsinki, Helsinki, Finland

**Keywords:** trans-generational immune priming, trade-offs, American Foulbrood (AFB), honeybee (*Apis mellifera* L.), costs

## Abstract

Honeybees are major pollinators for our food crops, but at the same time they face many stressors all over the world. One of the major threats to honeybee health are bacterial diseases, the most severe of which is the American Foulbrood (AFB). Recently a trans-generational vaccination approach against AFB has been proposed, showing strong potential in protecting the colonies from AFB outbreaks. Yet, what remains unstudied is whether the priming of the colony has any undesired side-effects. It is widely accepted that immune function is often a trade-off against other life-history traits, hence immune priming could have an effect on the colony performance. In this experiment we set up 48 hives, half of them with primed queens and half of them as controls. The hives were placed in six apiaries, located as pair of apiaries in three regions. Through a 2-year study we monitored the hives and measured their health and performance. We measured hive weight and frame contents such as brood amount, worker numbers, and honey yield. We studied the prevalence of the most common honeybee pathogens in the hives and expression of relevant immune genes in the offspring at larval stage. No effect of trans-generational immune priming on any of the hive parameters was found. Instead, we did find other factors contributing on various hive performance parameters. Interestingly not only time but also the region, although only 10 km apart from each other, had an effect on the performance and health of the colonies, suggesting that the local environment plays an important role in hive performance. Our results suggest that exploiting the trans-generational priming could serve as a safe tool in fighting the AFB in apiaries.

## 1. Introduction

Majority of the plant species used as food are dependent on pollinators, and it is estimated that by production volume 35% of our crops depend on pollinators, like wild and managed bees (1). Alarmingly the numbers of pollinators have been in decline (2, 3). Not only is the decline seen in wild pollinators, but the health of managed bee colonies has also declined, with reported yearly losses typically 15–30% in Europe and the US (4, 5). These losses are caused by multiple factors, including changes in landscape use, pesticides, parasites, and pathogens (6). As pathogens are among the many reasons behind the declining honeybee health, understanding the insect immune system and resistance mechanisms is a key for finding ways to combat honeybee colony losses. As managed honeybees and wild pollinators may share diseases and parasites (7), improving the health of managed honeybees may also improve the health of pollinators in general.

Immune priming is a function of the invertebrate immune system that can improve resistance against a previously encountered pathogen. The priming effect can also extend to the next generation (8) and is then called trans-generational immune priming (TGIP). Mechanisms of TGIP are still being investigated and proposed mechanisms include epigenetic modifications (9) and transfer of immune reaction elicitors or the effector molecules themselves into the developing eggs (10, 11). There is still a level of controversy around TGIP in different insect species, as mixed results concerning TGIP have been reported using different hosts, different pathogens, or ways of exposure. Some hosts may show TGIP against certain pathogens, while not against others (12), suggesting that there may be differences due to exposure route, genetic or epigenetic factors of the host, or the type of pathogen. In honeybees a similar situation exists, as experiments with TGIP against viral pathogens have had mixed results (13, 14). Similarly mixed results have been found with bacterial pathogens. Recently a study found no evidence for TGIP in honeybees against a bacterial pathogen European Foulbrood (*Melissococcus plutonius*) with oral priming (15), while other studies have shown TGIP against other bacterial pathogens using either oral (16) or injection route of priming the honeybee queen (17).

Increased resistance to a pathogen would be beneficial, but immune system activation tends to be costly (18). Costs of pathogen resistance may be seen in tradeoffs with other life-history parameters, such as reproduction or growth. In insect hosts, a cost of priming has been seen as reduction in egg hatching rate in the mosquito *Anopheles albimanus* (19), but also as reduced fecundity of the offspring in the tobacco hawk moth (*Manduca sexta*) and the red flour beetle (*Trilobium castaneum*) (20, 21). In the mosquito *A. gambiae*, yellow mealworm beetle (*Tenebrio molitor*), red flour beetle and tobacco hawk moth an increase in offspring developmental times have been observed (22–25), although faster offspring development has been observed as well in tobacco hawk moth and *Trilobium* beetles (21, 26). In Hymenoptera particularly, TGIP have been studied with multiple models including ants, bumble bees and honeybees (17, 27, 28). In bumble bees, distinct costs of TGIP were seen as the parents produced less offspring, and while the offspring were more protected against the focal pathogen, they were more susceptible to other unrelated pathogens (29).

Honeybees have been used as model species for TGIP in multiple studies (10, 11, 13–17, 30) and TGIP has been shown to be effective against one of the most devastating honeybee diseases worldwide, American Foulbrood (AFB), caused by the spore-producing bacterium *Paenibacillus larvae* (31). AFB infects honeybee larvae after an oral exposure, and infection requires the permeation of the peritrophic membrane in the gut (32). Infected larvae die shortly after, becoming sources of exposure themselves. Spores of AFB can stay viable and attached to the affected beekeeping equipment for decades (31). An acute infection therefore requires the destruction of the hive boxes and anything that has been in contact with the hives, typically by burning, causing also considerable financial burden on beekeepers of the affected hives. Solutions to limit AFB are limited, relying mostly on good beekeeping practices and in some regions also antibiotics (33). TGIP has been suggested as a way to combat diseases such as AFB in honeybees (30), but the knowledge on costs of TGIP on honeybees' health is limited.

In this study we investigate the effects of trans-generational immune priming on honeybees using AFB as the priming agent, in a typical beekeeping setting. We used dead *P. larvae* to orally prime honeybee queens and followed the hives for two seasons. Given the multitude of observed potential costs and tradeoffs in other model species, we took multiple approaches to study the TGIP effects on a colony level, assessing hive health parameters, pathogens in the hive, and gene expression in offspring. If there would be immune priming costs associated with egg laying, hatching rate or brood development, it should translate into hive size or the ability of the hive to forage and collect honey. As shown in bumblebees, a potential tradeoff of TGIP is the susceptibility of the offspring to other pathogens (29). To describe the pathogen load of the bee colonies, pathogens and parasites were assayed, representing the most common bacterial, viral and fungal pathogens infecting honeybees. To study the consequences of TGIP on a molecular level we did a targeted RT-qPCR assay on genes relevant to TGIP or pathogen defense in developing larvae. Differences in gene expression would be expected if TGIP is mediated to the next generation through elicitors of immune responses or through epigenetic modification. We targeted genes involved in immunity, TGIP, and AFB responses identified in the literature.

To summarize, the focus of our study was to investigate whether there are tradeoffs from TGIP in honeybees in a typical beekeeping setting. Based on current evidence on effects of TGIP, we expected to see differential gene expression in the offspring and potential costs manifesting in higher susceptibility to other pathogens or as decreases in hive size or productivity.

## 2. Materials and methods

### 2.1. Honeybees and queen treatment

In this study we examined honeybees of the subspecies *Apis mellifera ligustica*. Naturally mated sister queens (*n* = 29 control, 28 primed, 57 total), inspected to successfully lay eggs, were orally primed by placing them in queen cages along with a small number of worker bees (6–11) and a bee candy patty, which the workers feed to the queen. The priming treatment bee candy included heat killed *P. larvae* bacteria. Control queens received a bee candy patty without the bacteria. Queen cages were kept over moist sponges at room temperature for 6 days to allow consumption of the candies. Survival of the queens and attending worker bees was monitored on a daily basis.

### 2.2. Preparation of queen feed

Priming treatment bee candy was prepared by mixing 5 g powder sugar, 500 μL corn syrup and 300 μL *P. larvae* solution, resulting in 1.5^*^10^8^ *P. larvae* cells per candy. Control candies were made the same way but using water instead of *P. larvae* solution. The *P. larvae* solution was made by growing *P. larvae* (strain LMG 09820 from Belgian Coordinated Collections of Microorganisms, Belgium) on MYPGP agar plates (34) in 34.5°C for 3 days and the bacteria were harvested into cold water. Cell concentration was measured by optical density with NanoDrop One C (Thermo-Fisher, USA), and the bacteria was killed by autoclaving at 121°C for 15 min. Autoclaved solution was again plated on MYPGP plates to confirm the lack of viability.

### 2.3. Hive and apiary establishment

Forty-eight Langstroth-type hives were established for the experiment. Worker bees used for the hives were collected from hives in three different locations, serving as founding stocks, and placed in hive boxes with five new empty frames to establish the new hives. The treated queens in their cages were then placed in these hives created with 3 l of adult bees, approximating about 10,000 bees (35). The new hives were placed in cool (~+15°C) and dark conditions for 48 h to accommodate, after which they were placed outside in a temporary apiary location and were left there for 2 weeks to avoid further stress, before transportation to six apiaries. Four primed hives and four control hives were placed in each apiary in alternating pattern such that every second hive has the priming treatment.

**Figure 1:**
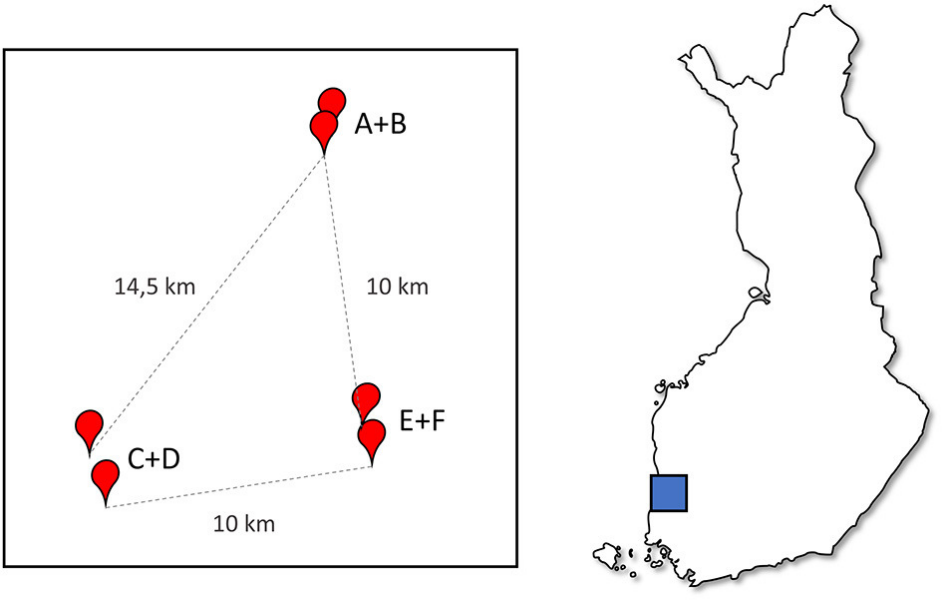
A diagram of the apiary locations relative to each other, apiaries (A–F) marked with red points showing distances, and the location in Finland (map box size arbitrary).

The apiaries were located in three regions in the Southwest Finland, two apiaries in each region (Figure 1). The apiaries are here called A-F, while the regions are named AB, CD, and EF, based on the two apiaries within the region. The regions were c.a. 10 km away from each other, which is more than the typical flying distance of a honeybee (36). In each region the two apiaries were close to each other, 0.5 to 2 km apart, within the common flying distance of bees. During the experiment the hives were maintained by two experienced beekeepers in a similar fashion using conventional beekeeping practices in Finland, using oxalic acid for Varroa-treatment (37) and with the exception that weak hives were not artificially strengthened or combined. The beekeepers were blinded regarding the priming treatment.

### 2.4. Hive assessments and sampling

**Figure 2:**
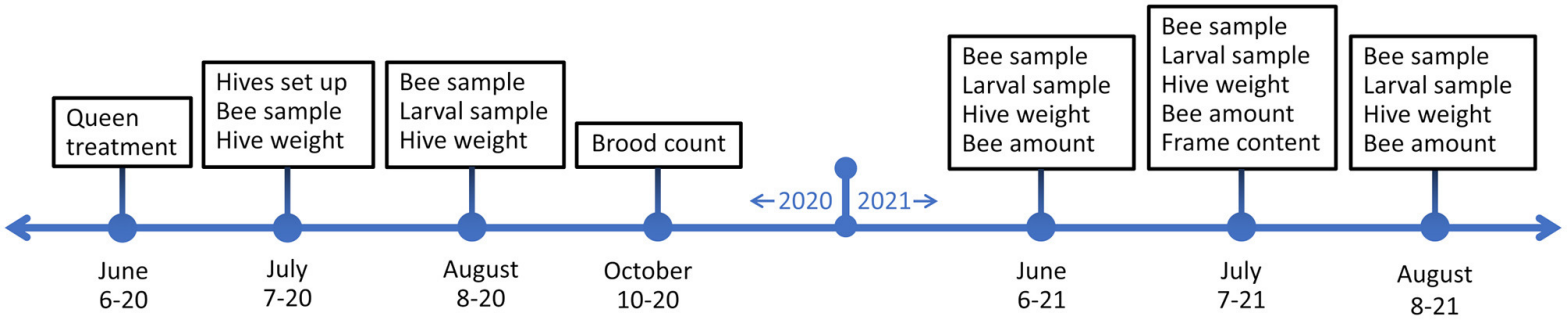
Experiment timeline and schedule of sampling times and hive measurements.

Hives were sampled and measured at five points in the summers of 2020 and 2021 (Figure 2). An initial sampling was done after the hives were set up in July 2020 (18.07.2020) and then the first full sampling in August 2020 (10.8.−13.8.2020). In 2021 sampling was done in June (10.6.−12.6.2021), July (10.7.−14.7.2021), and August (10.8.−13.8.2021). Hive weight was measured, and bee samples for pathogen quantification were taken in all sampling times and during hive setup. Larval samples for gene expression were taken at all sampling times after hives were set up. Bee amounts were estimated during 2021 samplings. Frame contents were measured during the July 2021 sampling. In addition, brood count was measured before winter in 2021, in October.

Queen survival and brood production (brood surface area) before winter were used to assess the potential costs of the priming for the queen. The amount of brood was measured by placing a 4x5-square grid over the brood frames and estimating the brood covering each frame, calculating the percentage covered. The development of the hives was followed by measuring the hive weight and the number of worker bees. Hive weight is a crude but often used and very effective method for following the development of the beekeeping season in terms of nectar flow into the hive (38). Weight measurements were done by weighing each box in the hive separately with a digital luggage scale (Asaklitt, Sweden), to the accuracy of 100 grams. The bee amounts were measured in 2021 visually from the frames from above, counting the gaps between frames that were fully and half occupied by bees. During the peak flowering season in Finland, in July 2021, a more detailed hive analysis was made to each hive. The contents of every frame were measured by placing a 4x5-square grid over the frame and estimating the amounts of grid squares filled with different types of content. The contents were assigned to categories of uncapped brood, capped brood, honey, nectar, pollen, and drone cells. After the samplings in the fall of 2021 the weight of the extracted honey was measured by weighing the boxes before and after extraction. Honey was extracted from the frames by the beekeepers using a centrifugal honey extractor.

To quantify the pathogens in the hives, adult bees were sampled. Adult bees of undefined age were collected from inside the hive by brushing from the frames (39). One dL of bees, equaling to ~300 bees per hive, were collected into a resealable plastic bag which was immediately placed on dry ice. Collected samples were briefly stored in −20°C, transported to laboratory on dry ice and stored in −80°C.

To investigate the gene expression of the larvae, 3-day-old larvae were sampled from hives at four timepoints, in August 2020 and thrice in 2021 for the gene expression assay. Three replicates of 10 larvae were collected from each hive. Ten larvae were collected from three frame surfaces, storing each 10 larvae into a microcentrifuge tube filled with 500 μL of NucleoProtect-RNA (Macherey-Nagel, Germany), in total primed *n* = 39^*^3 = 117, control *n* = 37^*^3 = 111.

### 2.5. Pathogen quantification

To assess the presence of pathogens in the hives, we targeted 10 common honeybee pathogens. Two bacterial pathogens were targeted, *Paenibacillus larvae*, which causes American foulbrood and *Melissococcus plutonius*, which causes European foulbrood (EFB). Viral targets were Acute Bee Paralysis Virus (ABPV), Black Queen Cell Virus (BQCV), Chronic Bee Paralysis Virus (CBPV), Deformed Wing Virus (DWV-B), and Sacbrood virus (SBV). Fungal pathogens were Chalkbrood (*Ascosphaera apis*), and the two microsporidian *Nosema* species, *N. apis* and *N. ceranae*. These pathogens have global distribution and are commonly found in Europe (40–44). In addition, we monitored the occurrence of Varroa mite (*Varroa destructor*), which is linked to decreased health of parasitized hives as well as spread of bee viruses (45, 46).

To quantify these pathogens, three sets of 33 bees were picked from the collected bee sample for RNA extraction, resulting in three biological replicates from each hive (primed samples *n* = 87, control samples *n* = 90, each sample with three replicates). The bees were visually inspected for *Varroa* mites before extraction (35). RNA was extracted following the bulk extraction protocol by Evans et al. (47), with the modification of freezing the sample in liquid nitrogen after adding the lysis/stabilization buffer. Extraction was quantified with NanoDrop One C (Themo-Fisher, USA) and samples stored at −80°C.

Prior to qPCR-step the RNA samples extracted above were treated with DNase I, RNase Free kit (Thermo-Fisher, USA) and reverse transcribed using RevertAid First Strand cDNA synthesis kit (Thermo-Fisher, USA), using manufacturer protocols. SsoAdvanced Universal SYBR Green Supermix (Bio-Rad, USA) was used for qPCR reactions with the manufacturer's protocol for 10 μL reaction volume, using CFX384 thermal cycler (Bio-Rad, USA). All reactions were run as two technical replicates. Ten pathogen targets given above were used, along with two reference genes, Actin and RPS18, which show stability under different conditions (48). Primer sequences were obtained from literature (Supplementary Table 1).

### 2.6. Larval gene expression

To assess the gene expression in the offspring, we targeted seven genes: *apidaecin, hymenoptaecin, PEPCK, peritrophin, PGRP-LC, PPO*, and *trynity*. *Apidaecin* and *hymenoptaecin* are antimicrobial peptides (AMPs), representing the end products of immune pathways. AMPs are upregulated after TGIP and infection (49, 50), indicating an immediate readiness for defense. *PEPCK*, stress-responsive phosphoenolpyruvate carboxykinase, is a gene involved in gluconeogenesis, and is upregulated after TGIP and infection in bumble bees, acting as a potential marker for TGIP (50). *Peritrophin* supports gut integrity, and as AFB requires the permeation of the gut membrane, *peritrophin* upregulation may act as an AFB specific defense response (51). *PPO*, prophenoloxidase, is the precursor for phenoloxidase, involved in the melanization immune reaction. Upregulation of *PPO* would indicate increased infection response readiness (52). *PGRP-LC* is a membrane bound peptidoglycan recognition protein, having a major role in pathogen recognition in honeybees (49). Upregulation of *PGRP-LC* would indicate increased readiness to recognize pathogens and to activate the particular immune pathways. *Trynity* is a zona pellucida domain protein, also expressed in the gut, and is upregulated in honeybee larvae after an AFB infection, indicating a potential AFB-specific response or defense mechanism (51).

To assess the expression of the selected genes, RNA was extracted from the larval samples with RNAzol (Sigma-Aldrich, USA). The samples, stored in 1.5 mL microcentrifuge tubes in−20°C, were thawn and quickly centrifuged. Storage buffer was removed, 1 mL of RNAzol was added, and the manufacturer protocol for total RNA isolation was followed. Extraction was quantified by NanoDrop One C (Thermo-Fisher, USA) and the samples were stored in −80°C. RT-qPCR for the larval gene expression assay was performed using the same procedure as in pathogen RT-qPCR. Same reference genes were used as in pathogen assay, Actin and RPS18. Other primers were implemented from literature (Supplementary Table 2).

### 2.7. Statistical analyses of queen failures and hive assessments

Queen failures were analyzed with a cox survival regression model. Treatment and apiary were included as fixed variables. Models with region, beekeeper, and worker stock site were excluded after considering AIC and BIC. For hive measurements the model selection was done similarly by comparing models with explanatory variables, which were apiary, region, beekeeper, and worker stock site. Model selection was based on AIC, BIC, and model *R*^2^. Region was chosen as the fixed variable for the peak season detailed frame content measurement in August 2021, and total honey yield models. Apiary was chosen for the hive weight and bee amount models, with apiary-timepoint interaction, and hive was used as random variable. The priming treatment was always kept in the model as fixed variable. Capped brood and open brood, as well as honey and nectar were combined for the peak season frame content models.

### 2.8. Statistical analyses for pathogen quantification and gene expression

A ddCq-approach was taken to analyze the pathogen prevalence and abundance in adult bees, measured by qPCR, as per the 2^−ddCq^ method (53). The Cq values of the two reference genes were averaged with geometric mean and this value was used for delta-Cq calculations. Missing dCq values were substituted with a value one cycle higher than the detected maximum value (54). The replicate values were then averaged. The dCq values were calibrated by using the highest value for each pathogen as the reference (sample dCq—max dCq), resulting in the ddCq values. For easier interpretation, the models and figures were done with negative ddCq values, corresponding to log_2_(2^−*ddCq*^). A higher value then indicates higher pathogen prevalence. Pathogen model selection was done similarly to hive measurement models. Models with the grouping factors apiary, region, beekeeper, and worker stock site were compared with AIC, BIC, and model *R*^2^, and the best performing models were used, keeping hive as a random factor. For AFB and Chalkbrood, a ddCq model could not be properly fitted due to low number of detections in the samples. A binomial presence-absence model was instead used with hive as random factor. No model was used for CBPV, ABPV, and EFB due to lack of data.

Gene expression in the larvae was analyzed using dCq values, which were derived in the manner described above, except no substitutions were needed for the gene expression dCqs. Negative values were again used for models and figures for easier interpretation. The gene expression model selection followed similar method as all the models described above. Region was selected as the best explanatory variable for all of the models, with treatment and timepoint kept as explanatory factorial variables and hive as a random factor. To include potential effects from pathogens, the models were tested with all pathogen dCqs as variables. Other pathogens were excluded due to lack of data. The pathogens that had significant effects were kept in the model. Some models yielded random effect variances that were close to zero, but the structure was kept in all of the models for consistency.

To study pathogen effects on other hive characteristics, we used the pathogen ddCq values in August 2020, the timepoint before overwintering. Pathogen effect on queen failures was analyzed with cox survival regression with apiary and all of the pathogens as fixed factors. Mixed effect models were used for hive weight, bee amount, and frame content measurements. For these models the treatment was dropped as a factor. No pathogens were indicated as having a significant effect.

Hives that were lost due to queen failure were excluded from the analyses after the point of failure. All analyses were implemented with R version 4.0.4 (55). Linear mixed models were done using the lme4-package (56) with significance testing using lmerTest (57). For the cox regression model, package survival (58) was used. The chosen models were inspected with DHARMa-package (59) and sjPlot was used to extract model information (60).

## 3. Results

### 3.1. Queen treatment and hive failures

No queens died during the priming period of 6 days in the queen cages. On average 0.84 (SD 1.33) accompanying workers died during the queen treatment, being no different between treatments (χ^2^ = 0.30905, *p* = 0.58). During the hive establishment four control queens and one primed queen were replaced due to not being accepted by the hive. This did not affect initial hive numbers since there were surplus queens in the priming treatments (29 primed and 28 control) than were used to create the hives (*n* = 48) to account for initial losses.

**Figure 3:**
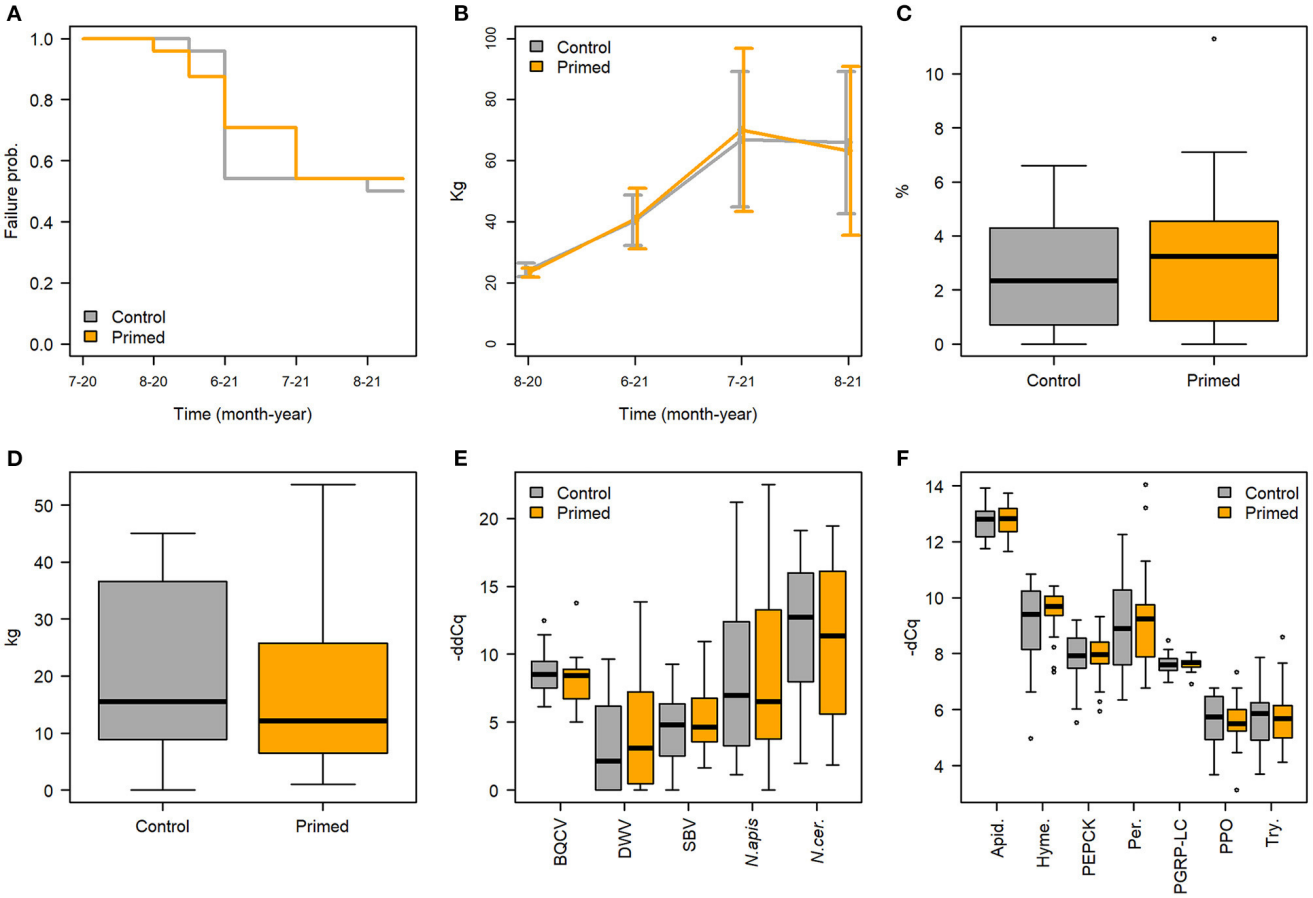
No effect of priming was detected to hive performance and health in regard to **(A)** hive failures, **(B)** median hive weight (+-sd), **(C)** percentage of brood on frames before winter, **(D)** overall honey yield, **(E)** pathogen -ddCq in August 2020, **(F)** gene expression -dCq in August 2020. Primed hives shown in orange and controls in gray.

There was no difference in hive failures during the experiment between the priming and control treatments (Figure 3A, Cox regression model LR-test *p* = 0.03, treatment HR = 1.08, *p* = 0.85), while apiary had a significant overall effect (*p* = 0.016) on hive survival. Two control and two primed hives died during the winter 2020–2021. Otherwise hive failures were relatively frequent during the experiment. In some cases, the queen started to lay only unfertilized eggs, becoming drones, during the spring 2021, soon leading to death of the hive. In other cases, the hive had raised a new queen for no apparent reason. Any hive without a queen or a queen replaced by the colony was considered as a queen failure and excluded from the experiment. Altogether queen failures took place in 19 hives, which is about 40% of hives, of which 10 were control hives and nine primed hives. Overall, out of 48 initial queens, after winter mortality and queen failures, 25 hives were left to be observed through the whole experiment (Table 1). In each apiary one to six hives faced a failure during the experiment, apiaries B, C, and D having a significant effect on the queen failure (Supplementary Table 3).

**Table 1:** Number of queens failing due to different causes during the experiment in each apiary and the treatment (control/priming).

	**Winter mortality**	**Drone queen**	**Queen replaced**
Apiary A	0/0	0/1	0/0
Apiary B	0/0	0/1	3/2
Apiary C	0/1	2/1	0/2
Apiary D	1/0	2/2	0/1
Apiary E	0/0	0/0	1/0
Apiary F	1/1	0/0	1/0

### 3.2. Hive assessment

The priming treatment did not significantly affect hive weight (Figure 3B, Supplementary Table 4), the amount of brood on the frames before winter (Figure 3C, Wilcoxon rank sum test, W = 261.5, *p* = 0.59), honey production, as measured by weight of extracted honey (Figure 3D), or the number of bees in the hives (Supplementary Table 5). No differences were also observed between treatments in the frame contents during the peak season detailed measurements in July 2021 (Supplementary Table 6).

**Figure 4:**
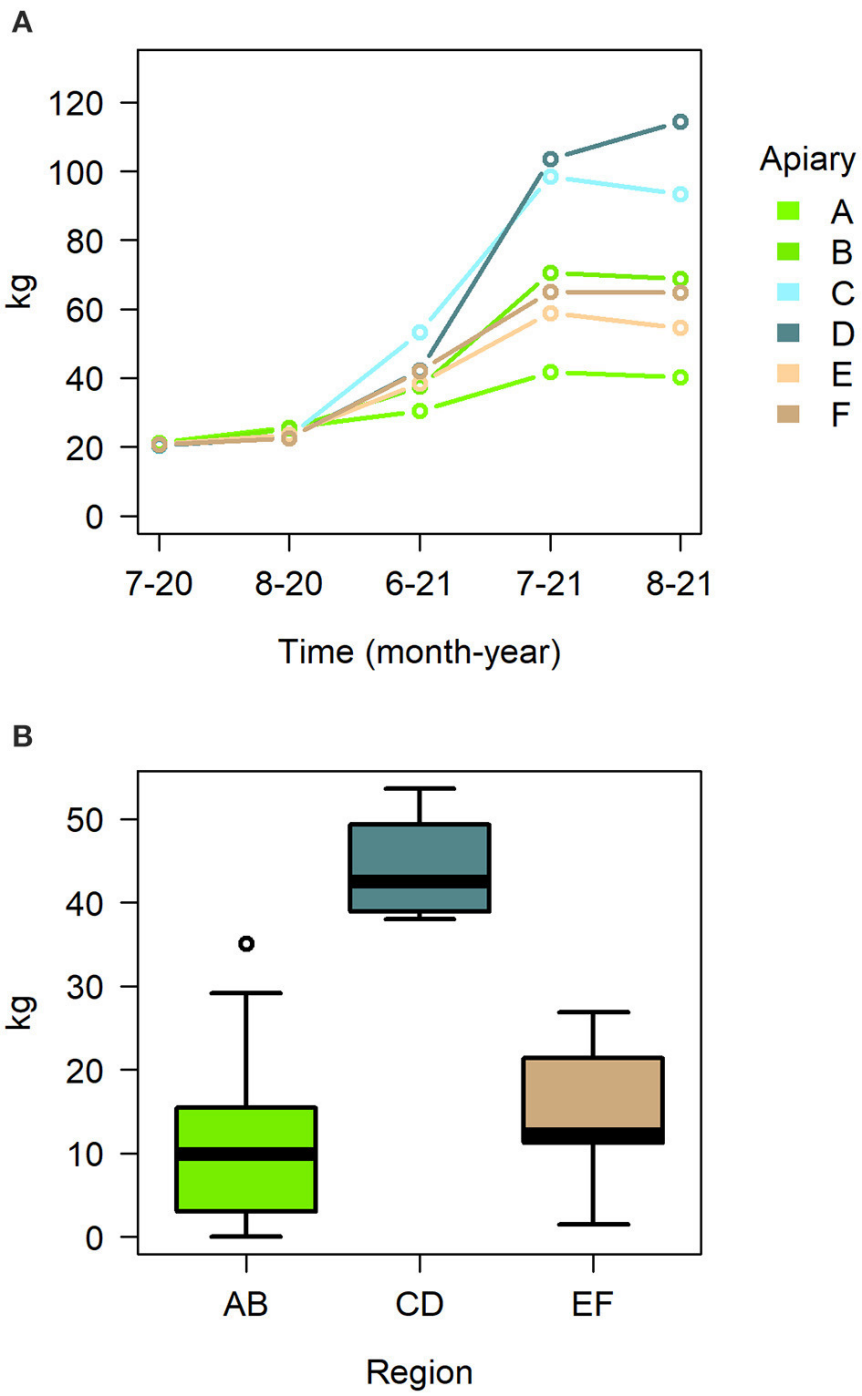
**(A)** Median hive weight in apiaries during the season 2021, **(B)** honey yield in the different regions as boxplot with mean and quartiles.

While treatment had no effect on any of the hive metrics, the time of the season and the location of the hive did have an effect on several metrics (Table 2). The linear mixed model on hive weight indicates apiary and sampling time to have a significant effect, as well as their interactions (Figure 4A, Supplementary Table 4). Adult bee amounts were overall significantly affected by time (Table 2), but the linear model indicated only apiary C as significant effect (Supplementary Table 5). Region had a significant overall effect in the linear models of brood, honey and nectar and drone cell amounts in the peak season detailed hive measurements in July 2021 (Table 2). The region CD had a significant effect on the cover of honey, pollen, and drone cells, and the region EF on honey and pollen, each being higher in these regions (Supplementary Table 6). There were also significant differences between regions on honey yield, the region CD doing significantly better (Figure 4B, Supplementary Table 7).

**Table 2:** Summary table of significant effects in the models reported in this study, as indicated by Type II/III analysis of variance tables (Satterthwaite's method).

		**Priming treatment**	**Time**	**Apiary**	**Region**	Location *time interaction	**Worker source**	**BQCV**
Health and performance	Survival	-		**				
	Honey yield	-			***			
	Peak brood	-			*			
	Peak honey	-			***			
	Peak pollen	-			**			
	Peak drones	-			*			
	Weight	-	***	***		***		
	Bees	-	***	-		-		
Pathogen prevalence	AFB	-	***				**	
	Chalkbrood	-	-		-			
	*N. apis*	-	*	-		***		
	*N. ceranae*	-	***	***		***		
	BQCV	-	***	***		***		
	DWV	-	***	***		***		
	SBV	-	***	-		**		
Gene expression	*Apidaecin*	-	***		***	*		*
	*Hymenoptaecin*	-	***		***	*		
	*PEPCK*	-	***		***	-		
	*Peritrophin*	-	-		-	-		
	*PGRP-LC*	-	-		***	***		*
	*PPO*	-	**		***	*		
	*Trynity*	-	***		-	-		

Location refers to apiary or region, depending on which one was used in the model. Significant effects indicated with stars (^*^p < 0.05, ^**^p < 0.01, ^***^p < 0.001), variables included in the model with no significant effect indicated with dash (-).

### 3.3. Pathogen prevalence

The study hives showed a complex pattern for the presence and absence of different pathogens. Surprisingly, not a single sample was positive for European Foulbrood (*Melissococcus plutonius*) or Acute Bee Paralysis Virus. Chronic Bee Paralysis Virus was detected in hives only 4.5% of times. American Foulbrood (*Paenibacillus larvae*) was detected in hives 14.1%, and Chalkbrood (*Ascosphaera apis*) 31.6% of times. Deformed Wing Virus was detected 85.6%, *Nosema Apis* 96.6% and *Nosema Ceranae* 86.4% of times. Black Queen Cell Virus and Sacbrood Virus were detected at all times in every hive.

The data for CBPV was too scarce to fit an informative model. Among the grouping variables tested, apiary was the best explanatory variable for the -ddCq-models, and apiary-timepoint interaction was included. For AFB the worker stock source location was the best explanatory grouping variable instead of apiary for the AFB binomial model (Supplementary Table 8). There were no positive AFB samples after June 2021 and the latter sampling timepoints were therefore excluded from analysis. For Chalkbrood a binomial model was also fitted with the region as explanatory variable.

**Figure 5:**
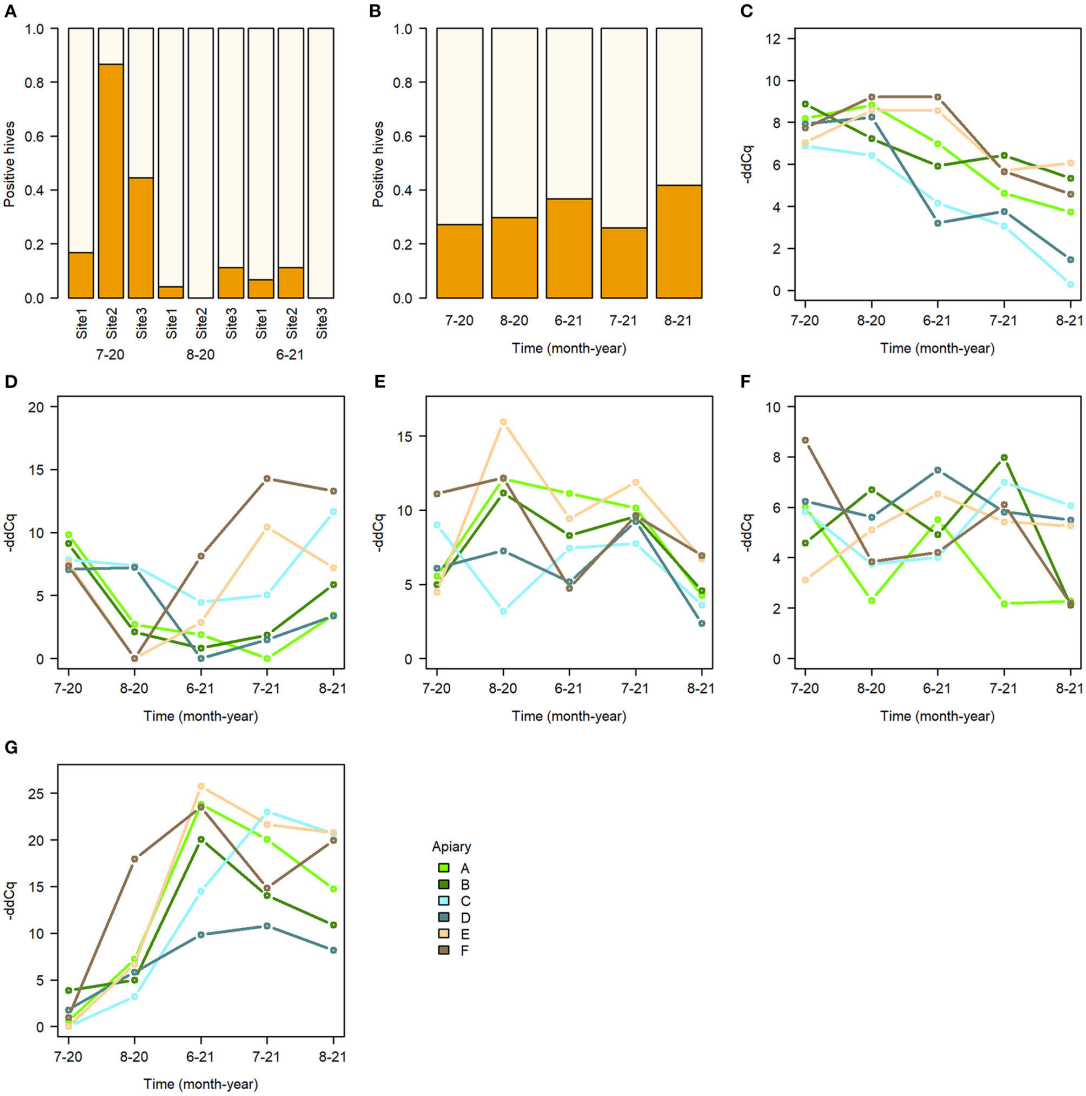
Summary of pathogens in sampled bees. **(A)** Proportion of AFB detected in hives with bees from different worker stock sites, **(B)** proportion of Chalkbrood detected in hives, **(C–G)** median pathogen -ddCq values in apiaries. **(C)** BQCV. **(D)** DWV. **(E)** SBV. **(F)** *Nosema apis*. **(G)** *Nosema ceranae*.

None of the pathogen models indicated treatment as significant effect (Table 2, Figure 3E). However, spatial and temporal effects were evident. For AFB, the model indicated worker stock source site 1 as significant effect (Figure 5A, Supplementary Table 8), for the sampling times August 20 and June 21. The model for Chalkbrood indicated that August 2021 had a significant effect but had no significant spatial effects (Figure 5B), although the model overall had limited explanatory power (Model *R*^2^ = 0.063, Supplementary Table 9). The dynamics for the viral pathogens differed from each other. BQCV model indicated July-21 and Aug-21 as significant effects, as did several time-apiary interactions (Figure 5C, Supplementary Table 10). A similar trend toward lower BQCV loads is observable over time in all apiaries (Figure 5C). For DWV every sampling time had significant effect, as had Apiaries C, D, and F, with several time-apiary interactions also significant (Figure 5D, Supplementary Table 11). Sacbrood virus had inconsistent dynamics between apiaries and sampling times, the model indicating sampling times Aug-20, June-21, and July-21 and apiaries C and F having significant effects, and several time-apiary interactions (Figure 5E, Supplementary Table 12). The two *Nosema* species also showed different dynamics from each other. *Nosema apis* levels were overall more consistent (Figure 5F, Supplementary Table 13), while *N. ceranae* levels showed more variation but similar trends in all apiaries, the levels being lowest in the initial sample in July 2020, and highest in June 2021 (Figure 5G, Supplementary Table 14).

In addition to pathogens, the occurrence of Varroa mites on the bees was measured. Overall, the mite count was very low, as only eight mites were seen attached to the bees during the whole study, five in control and three in primed hives (Wilcoxon rank sum test, W = 3,963.5, *p* = 0.45).

### 3.4. Gene expression

**Figure 6:**
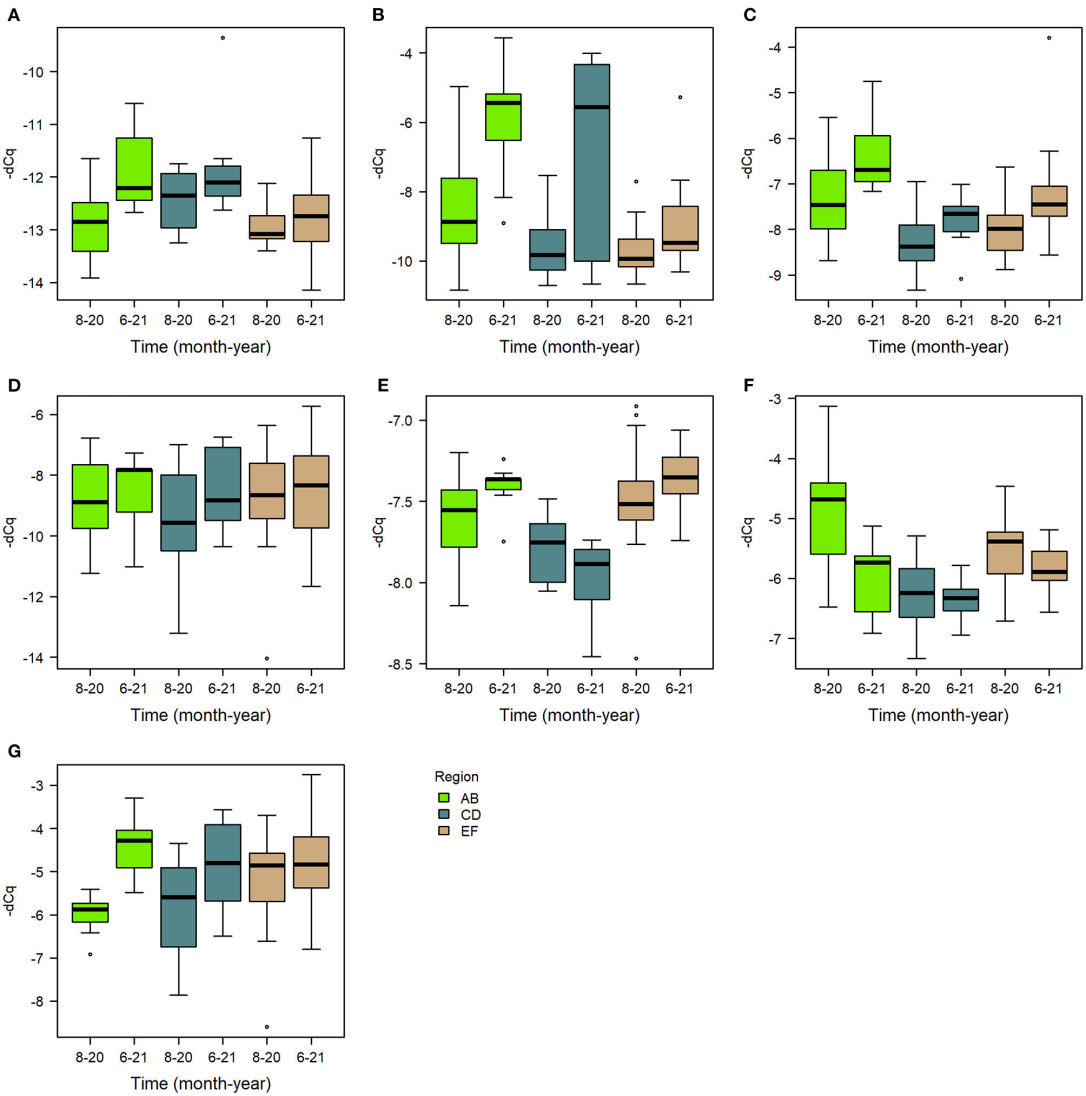
Boxplots of gene expression levels of the assayed genes **(A)** *apidaecin*, **(B)** *hymenoptaecin*, **(C)** *PEPCK*, **(D)** *peritrophin*, **(E)** *PGRP-LC*, **(F)** *PPO*, and **(G)** *trynity*, in August 2020 and June 2021 in each region.

The priming treatment did not affect the expression of any of the studied genes (Figure 3F, Supplementary Tables 15–21). Instead of treatment, the sampling time had a clear effect on gene expression. All genes except for *PER* and *PPO* had a higher expression in June 2021 than in the previous August 2020 (Figure 6). Differences were seen also by region (Table 2), and in some cases the black queen cell virus had an effect. As no treatment effects were observed in the first two timepoints, further samples were not processed. In *apidaecin* and *hymenoptaecin* a similar trend was observed, with expression being higher in every apiary region in the June-21, the effect in both being smallest in the region EF (Figures 6A, B, Supplementary Tables 15, 16). For *Apidaecin* there was also a modest negative correlation with BQCV (Supplementary Table 15). The absolute levels in *PEPCK* expression differ between regions, but the general trend also is a higher expression in June-21 (Figure 6C, Supplementary Table 17). In *peritrophin* no significant effects were found (Figure 6D, Supplementary Table 18), although the model as a whole had very limited explanatory power (model *R*^2^ 0.055). *PGRP-LC* expression had the least variation between the two times and across apiary regions, the linear model indicating slight increase in expression in June 2021 (Figure 6E, Supplementary Table 19). In addition, BQCV had a modest effect increasing *PGRP-LC* expression (Supplementary Table 19). *PPO* expression in June 21 was overall lower than in August 2020, with also regions and time-region interactions having significant effects (Figure 6F, Supplementary Table 20). *Trynity* expression was again significantly higher in June 21, but regions did not have significant effects (Figure 6G, Supplementary Table 21).

### 3.5. Effects of pathogens on other hive characteristics

Pathogens in the adult worker bees in August 2021 did not have a significant effect on the hive failures (Cox regression model LR-test *p* = 0.05, pathogen *p* > 0.05). The pathogens also did not have significant effects in any of the frame contents, hive weight, bee amount or honey yield (linear models, pathogen *p* > 0.05).

## 4. Discussion

In this study we found no negative effects of trans-generational immune priming on number of fitness-related traits in honeybees. The metrics used to evaluate the performance of the beehives are common for the beekeeping, such as hive weight, amount of brood, adult bees, and honey yield. Furthermore, TGIP also did not affect pathogens in the honeybees, nor gene expression in the larvae. Based on the TGIP literature, we expected potential tradeoffs in hive performance, pathogen presence and differences in offspring gene expression.

Tradeoffs in reproduction or brood development are common costs found in TGIP research (12). No study has specifically investigated brood development in honeybees under natural conditions, but evidence from other studies suggests that TGIP may come with the cost of longer offspring development times (12). While we did not directly measure brood development, an increase in offspring development times would slow hive growth and therefore indirectly influence hive weight and honey yield. Similarly, tradeoffs in queen reproduction would have resulted in less brood, meaning fewer adult bees and fewer foragers, which would also influence hive weight and honey yield. Yet, no such differences were observed in our study.

In bumble bees, the offspring of immune primed queens were better protected against the same pathogen, but more susceptible to other pathogens (29). It is possible that such tradeoffs also exist in honeybees, and in that case, we should see a higher presence of pathogens other than the priming agent in the primed bees. We measured several common honeybee pathogens, including bacterial, viral and fungal pathogens, but the priming treatment had no effect on any of them. Our results indicate that priming against AFB does not put hives in risk of other pathogens. The results are in agreement with another study in bumblebees, where a within-generation immune priming treatment similarly did not affect colony fitness or parasite infections (61). It is possible that costs of TGIP were compensated by the nutritional resources readily available in the environment. For example, organisms are typically able to compensate some of the infection costs by adjusting intake of required macronutrients (18, 62). Yet, in our study any compensatory nutritional needs of the honeybees due to TGIP did not translate to observable changes in the amount of pollen, honey yield or growth of the hives.

Multiple studies conducted in insects have shown TGIP induced changes in offspring gene expression. For example, the expression of immunity-related genes in red flour beetle eggs were markedly higher in offspring of primed mothers, including antimicrobial peptides (63). Similarly in bumble bee offspring an increase in the expression of immune system genes was shown, and the TGIP effect on gene expression seemed to be similar to primary infection (50). If this is the case in honeybees, we could also expect genes that are specific to AFB responses would be upregulated in offspring of queens primed with AFB. We therefore expected to see higher expression of genes that are induced in honeybees upon infection in general (49) and upon AFB infection specifically (51). However, we found no difference in the expression of the selected target genes between TGIP and control larvae. We targeted multiple genes in the immune system and other genes that were predicted to contribute to TGIP or have AFB specific effects. After seeing no priming effects on gene expression in the first two sampling timepoints, we decided to not process further samples, as the primary purpose of this study was to investigate effects of TGIP, and we found it unlikely that effects would be seen at later samples. Although there might be differences in different hosts, as honeybee gene expression after TGIP has been studied less than in other hosts, our assumption is that other uncontrollable environmental effects in our study were enough to mask the differences had they occurred. Interestingly the pathogen prevalence in the adult bees and immune gene expression in the larvae also did not correlate, as none of the pathogens had significant effects on the gene expression of any of the genes studied. Although priming was expected to lead to differential gene expression in offspring, in some cases the difference may not be evident until upon infection, if TGIP acts in biphasic manner, such that after initial infection the immune responses return to base levels until the pathogen is encountered again (64). In that case we would not expect to see different expression, particularly in genes that transcribe the end-products of immune responses, such as the antimicrobial peptides. On the other hand, differences could still have been seen in genes that confer readiness to respond to infections, such as pathogen recognition proteins.

Instead of predicted priming effects, we found many effects of the location and sampling time to be correlated with the hive parameters, underlying the importance of the environment for hive performance. Honey yield particularly was greatly affected by location. Hive weight also varied between apiaries, increasing over time in an expected manner. The amount of drone cells was the highest in the region CD. As the investment in reproduction is influenced by food availability (65), it is not surprising that the region with highest honey yield also had the most drones. The beekeeping practices were kept as conventional as possible, with the exception that weaker hives were not combined, a procedure that a beekeeper might commonly do to strengthen smaller hives (37). Therefore, the hive weights and honey yield might not perfectly reflect a typical situation. Differences in weight and yield per hive would be smaller between locations if weaker hives would have been allowed to be combined. Still, the hive weights reflect the typical Finnish beekeeping season, which often peaks in July.

Most of the pathogens targeted in this study were found in the hives. This is not unusual, as many bee pathogens, particularly viruses, are often present in low amounts in asymptomatic hives and only symptomatic hives have higher titers (66). No clinical symptoms were visually detected during the sampling and measurement of the study hives throughout the study period. The prevalence of pathogens in hives mostly followed typical seasonal dynamics, with peak prevalence in spring and summer (44), except DWV and BQCV. It is not uncommon, though, for the peak prevalence to shift from year to year (44). Another factor may be that the study hives were newly created at the beginning of the study, with the age of the hive and the bees playing a role, as the pathogens present in the hive are likely fewer when starting hives with fresh frames but may accumulate or find a more typical dynamic over time. Also, the initial reference sample consisted of bees from older hives, while bees in later samples were produced by the new queen. Especially interesting is that more AFB was detected in bees originating from one location, but then AFB practically disappeared and was not detected in any hive in later sampling times. New, presumably AFB-free frames and flame-treated hive boxes were used to create the hives, and therefore only the bees that were used to establish the new colonies were potentially carrying AFB spores. Although AFB spores elsewhere in the hive were not assayed, the fact that no AFB was detected in the bees at later times suggest that using clean hive equipment and replacing the queen has an impact on AFB pressure, highlighting the importance of good beekeeping practices (31). For pathogens other than AFB and chalkbrood the model selection strongly favored apiary as the grouping factor. This suggests that the immediate surroundings may have a stronger influence in the pathogen dynamics than for other aspects of the hive. Hives that are located next to each other are more likely to interact, especially since bees may sometimes enter other hives next to their own by accident or to rob resources. In this aspect it is not surprising that pathogen effects are localized to apiary level.

We saw higher gene expression in many of the studied genes in the June of 2021 than in August of the previous year. Mostly similar trends were observed in all of the regions. The antimicrobial peptides *Apidaecin* and *hymenoptaecin* were both less expressed in August than in the following June. Similarly, *PEPCK, PGRP-LC*, and *trynity* had lower expression in August 2020 than June 2021. The larval samples collected in mid-August are likely to be so called winter bees, whereas larvae sampled in the following June would be summer bees. Winter bees differ from summer bees by having substantially longer lifespan (67). Winter bees also differ in regards of gene expression, as adult winter bees show higher expression of *PPO*, but the expression of AMP genes is lower, perhaps as an energy saving measure (68). In our study AMPs were indeed lower in the fall, while *PPO* was higher. The differences between these two timepoints could then be partially explained by the differences in summer and winter bee gene expression profiles. We found that the pathogen BQCV affected the expression of *Apidaecin* and *PGRP-LC*, although the effect was modest. There was discrepancy in the samples however, as gene expression was studied in larvae and pathogens in adult bees. What we observed is that pathogen prevalence in adult bees is not very strongly correlated with immune gene expression in the larvae. The model selection for gene expression was not as strongly favoring any particular grouping factor as with pathogens, but overall region was the strongest. This suggests that environmental factors do play a role in the expression of many of the genes studied, but the effect is not as strongly localized as with pathogen prevalence, where apiary-level differences were stronger. It seems, that gene expression in the larvae is influenced also by other local, but not apiary-specific factors, like nectar and pollen availability or microclimate of the region.

We observed a relatively high rate of hive failures in our study, although it was not influenced by the queen treatment, as primed and control hives were similarly affected. Failure rates as high as 30% are not unheard of Gray et al. (5), although typically most hive failures happen during the winter season. Reasons for other observed queen failures are not all well-understood, but among the suspected causes are bad conditions during queen transport (69), exposure to pesticides (70) and problems during mating (71). The treatment conditions in our study mimic queen transport but were arguably more controlled than the varied conditions that the queens might be exposed to during transport in the mail. Further, similar methods used in other studies have not resulted in significant queen failures (13, 16). A likely reason for the queen failures then are problems during mating, such as bad weather conditions during mating or the drone quality. An important point regarding the actual hive survival is that not all of the excluded hives actually died. When the hive produces a new queen, the hive may continue to perform well, but in this study every hive without the original queen was excluded. Interestingly the failures did not occur equally among apiaries, but instead some apiaries were more affected. While the apiaries are located in the same geographical area, the immediate local environment surrounding the apiaries differ. The vegetation or buildings in the immediate surroundings of an apiary would affect the weather conditions the hives encounter, having an effect on the water and temperature regulation of a hive and thus on the resources a colony needs to use for the regulation (72). The apiary landscape would influence the hives by defining the availability of floral resources (73). Proximity to agricultural fields may also increase the risk of exposure to pesticides, which may compromise colony health (74). Overall, the microclimatic conditions, the resource availability and pesticide exposure are likely to contribute to local scale wealth of honeybees and may affect the survival of colonies in different apiaries.

This is the first study to investigate the effects of TGIP outside the laboratory under natural conditions with all the confounding environmental factors in an insect host. Our focus was to assess possible tradeoffs resulting from the priming treatment reported by number of laboratory studies in other species (19–26, 29). The strength of laboratory studies is the controlled environment and possibility to manipulate conditions to pinpoint even small effects, for example by inspecting tradeoffs under starvation, when tradeoffs may be more apparent. Studies outside of the laboratory are the necessary next step to see if tradeoffs or other effects are observable in the factual settings honeybees are kept in by beekeepers. Furthermore, as laboratory studies typically focus on individuals, colony level effects need to be considered in eusocial species, as done here.

To conclude, we could not find any significant trade-offs resulting from TGIP. These results have important practical applications, as TGIP has been recently suggested as a tool to fight infections by “vaccinating” honeybees against pathogens (16, 30). Our results support the use of TGIP as a tool in fighting American Foulbrood. In addition to the fact that TGIP has been shown to reduce AFB infections in honeybee larvae, our results provide evidence that TGIP also does not increase the load of pathogens not targeted by the priming treatment. There is also some evidence that viruses or other pathogens may spill over from managed honeybees to other pollinators (7, 75, 76). Using TGIP to reduce the pathogens in honeybee hives may therefore improve not only the health of honeybees but also the health of wild pollinators.

## Data availability statement

The original contributions presented in the study are included in the article/Supplementary material, further inquiries can be directed to the corresponding author.

## Author contributions

ML and DF designed the study. ML and HW collected the samples. ML ran the laboratory and statistical analyses and prepared the figures. ML, HW, and DL wrote the article. All authors contributed to the article and approved the submitted version.
