# Supplementary material for: Trans-generational immune priming against American Foulbrood does not affect the performance of honeybee colonies
Source: Front Vet Sci. 2023 Feb 27;10:1129701. doi: 10.3389/fvets.2023.1129701 (PMC10008890; doi:10.3389/fvets.2023.1129701)
Supplement: Supplementary file 1 [file Data_Sheet_1.docx]

Supplementary Material

Trans-generational Immune priming against American Foulbrood does not affect the performance of honeybee colonies

Matti Leponiemi*, Helena Wirta, Dalial Freitak

*** Correspondence:** Corresponding Author: [matti.leponiemi@edu.uni-graz.at](mailto:matti.leponiemi@edu.uni-graz.at)

# Data availability

The dataset used in this study can be found at Zenodo repository (DOI: 10.5281/zenodo.7470448)

# Primer information

Table S1. Primers used for pathogen quantification with NCBI accession number and reference.

| Name | Sequence | Accession # | Reference |
| --- | --- | --- | --- |
| Actin_F | AGGAATGGAAGCTTGCGGTA | AB023025.1 | (Chen et al., 2005) |
| Actin_R | AATTTTCATGGTGGATGGTGC |  |  |
| RPS18_F | GATTCCCGATTGGTTTTTGAATAG | XM_625101.6 | (Jeon et al., 2020) |
| RPS18_R | AACCCCAATAATGACGCAAACC |  |  |
| AFB_F | TTCGGGAGACGCCAGGTTA | CP019687.1 | (Rossi et al., 2018) |
| AFB_R | CTTTCATGACTTCTTCATGCGAAG |  |  |
| EFB_F | CAGCTAGTCGGTTTGGTTCC | EF666055.1 | (Roetschi et al., 2008) |
| EFB_R | TTGGCTGTAGATAGAATTGACAAT |  |  |
| AscoA_F | GGAATATGATCTGGTTTAGTTGGTC | U68313 | (D’Alvise et al., 2019) |
| AscoA_R | GAATCAATTTCCAAACCCACCAATC |  |  |
| NosemaA_F | CGTACTATGTACTGAAAGATGGACTGC | U97150.1 | (Huang and Solter, 2013) |
| NosemaA_R | AGGTCTCACTCTTACTGTACATATGTTAGC |  |  |
| NosemaC_F | GAGAGAACGGTTTTTTGTTTGAGA | U26533.1 | (Huang and Solter, 2013) |
| NosemaC_R | ATCCTTTCCTTCCTACACTGATTG |  |  |
| DWV_B_F | TATCTTCATTAAAACCGCCAGGCT | AY251269 | (Mcmahon et al., 2015) |
| DWV_B_R | CTTCCTCATTAACTGAGTTGTTGTC |  |  |
| ABPV_F | ACCGACAAAGGGTATGATGC | AF150629.1 | (VanEngelsdorp et al., 2009) |
| ABPV_R | CTTGAGTTTGCGGTGTTCCT |  |  |
| CBPV_F | CGCAAGTACGCCTTGATAAAGAAC | NC_010711.1 | (Blanchard et al., 2007) |
| CBPV_R | ACTACTAGAAACTCGTCGCTTCG |  |  |
| SBV_F | GGGTCGAGTGGTACTGGAAA | AF092924.1 | (VanEngelsdorp et al., 2009) |
| SBV_R | ACACAACACTCGTGGGTGAC |  |  |
| BQCV_F | GGTGCGGGAGATGATATGGA | AF183905.1 | (Chantawannakul et al., 2006) |
| BQCV_R | GCCGTCTGAGATGCATGAATAC |  |  |

Table S2. Primers used in gene expression assay with NCBI accession number, efficiency, R^2^ and reference.

| Name | Sequence | Accession # | E | R^2^ | Ref |
| --- | --- | --- | --- | --- | --- |
| Actin_F | AGGAATGGAAGCTTGCGGTA | AB023025.1 | 2,03 | 0,998 | (Chen et al., 2005) |
| Actin_R | AATTTTCATGGTGGATGGTGC |  |  |  |  |
| RPS18_F | GATTCCCGATTGGTTTTTGAATAG | XM_625101.6 | 1,95 | 1,000 | (Jeon et al., 2020) |
| RPS18_R | AACCCCAATAATGACGCAAACC |  |  |  |  |
| apidaecin_F | TTTTGCCTTAGCAATTCTTGTTG | GB17782 | 2,09 | 0,999 | (Evans et al., 2006) |
| apidaecin_R | GTAGGTCGAGTAGGCGGATCT |  |  |  |  |
| hymenoptaecin_F | CTCTTCTGTGCCGTTGCATA | NM_001011615 | 1,98 | 0,995 | (Cornman et al., 2013) |
| hymenoptaecin_R | GCGTCTCCTGTCATTCCATT |  |  |  |  |
| PEPCK_F | TGGTGGAATCTTTTGGGAAG | XM_396295.7 | 1,99 | 0,991 | (Jennette, 2017) |
| PEPCK_R | CTGGGGAACAGAATCTGGAA |  |  |  |  |
| peritrophin_F | GCAAACGACATTTCAATGGCAATCTTCAG | XM_003250167 | 2,01 | 0,997 | (Cornman et al., 2013) |
| peritrophin_R | CACATTGGTAATTGTATAGTACGTTCGCATC |  |  |  |  |
| PGRP-LC_F | TCCGTCAGCCGTAGTTTTTC | GB17188 | 1,88 | 0,995 | (Evans et al., 2006) |
| PGRP-LC_R | CGTTTGTGCAAATCGAACAT |  |  |  |  |
| PPO_F | GGACATCAATCGACAAGTTG | GB18313 | 1,97 | 0,999 | (Siebert et al., 2020) |
| PPO_R | GACGTCGATTCCATTTTTCT |  |  |  |  |
| trynity_F | GAGCGACGCTAGCATAGACTTC | XM_394451 | 1,97 | 0,999 | (Cornman et al., 2013) |
| trynity_R | GTGGAAATGTAGATGAGGACAAGCAG |  |  |  |  |

# Statistical model details

In the statistical model tables, apiaries are named A-F and the regions named after the two apiaries within the region (AB, CD and EF). In the models including time as a factor the sampling timepoints are named T0-T4, T0 corresponding to July 2020, T1 August 2020, T2 June 2021, T3 July 2021 and T4 August 2021.

## Details of hive assessment models

Table S3: Model details for cox survival regression model on queen failures throughout the experiment. Significant effects (p < 0.05) indicated with bold.

| Predictors | Estimates | CI | p |
| --- | --- | --- | --- |
| Treatment [priming] | 0.89 | 0.39 – 2.04 | 0.787 |
| **apiaryB** | **10.14** | **1.21 – 84.70** | **0.032** |
| **apiaryC** | **9.02** | **1.08 – 75.38** | **0.042** |
| **apiaryD** | **9.42** | **1.13 – 78.81** | **0.038** |
| apiaryE | 0.92 | 0.06 – 14.68 | 0.952 |
| apiaryF | 4.00 | 0.42 – 38.46 | 0.230 |
| Observations | 48 |  |  |
| R2 Nagelkerke | 0.295 |  |  |

Table S4: Model details for hive weight, with treatment, apiary, timepoint and apiary-timepoint interaction as fixed factors and hive as random factor. Significant effects (p < 0.05) indicated with bold.

| Predictors | Estimates | CI | p |
| --- | --- | --- | --- |
| **(Intercept)** | **30.68** | **18.40 – 42.96** | **<0.001** |
| Treatment [priming] | 4.45 | -5.46 – 14.36 | 0.372 |
| **T [T3]** | **13.86** | **5.85 – 21.86** | **0.001** |
| **T [T4]** | **11.90** | **3.89 – 19.91** | **0.004** |
| apiary [B] | 4.59 | -18.54 – 27.72 | 0.693 |
| **apiary [C]** | **18.34** | **0.26 – 36.42** | **0.047** |
| apiary [D] | 11.48 | -8.44 – 31.40 | 0.253 |
| apiary [E] | 7.70 | -7.24 – 22.64 | 0.306 |
| apiary [F] | 7.32 | -9.56 – 24.21 | 0.389 |
| **T [T3] * apiary [B]** | **19.19** | **2.21 – 36.18** | **0.027** |
| **T [T4] * apiary [B]** | **19.45** | **2.46 – 36.44** | **0.026** |
| **T [T3] * apiary [C]** | **34.76** | **20.31 – 49.22** | **<0.001** |
| **T [T4] * apiary [C]** | **31.98** | **15.77 – 48.19** | **<0.001** |
| **T [T3] * apiary [D]** | **43.69** | **27.00 – 60.38** | **<0.001** |
| **T [T4] * apiary [D]** | **56.50** | **39.80 – 73.19** | **<0.001** |
| **T [T3] * apiary [E]** | **13.59** | **2.63 – 24.56** | **0.016** |
| T [T4] * apiary [E] | 10.00 | -1.22 – 21.22 | 0.080 |
| T [T3] * apiary [F] | 12.18 | -0.22 – 24.59 | 0.054 |
| T [T4] * apiary [F] | 11.16 | -1.24 – 23.56 | 0.077 |
| Random Effects |  |  |  |
| σ2 |  | 56.08 |  |
| τ00 Hive |  | 151.64 |  |
| ICC |  | 0.73 |  |
| N Hive |  | 29 |  |
| Observations |  | 81 |  |
| Marginal R2 / Conditional R2 |  | 0.655 / 0.907 |  |

Table S5: Model details for bee amounts in hives, with treatment, apiary, timepoint and apiary-timepoint interaction as fixed factors and hive as random factor. Significant effects (p < 0.05) indicated with bold.

| Predictors | Estimates | CI | p |
| --- | --- | --- | --- |
| **(Intercept)** | **20.94** | **14.15 – 27.74** | **<0.001** |
| treatment [primed] | 1.11 | -2.75 – 4.97 | 0.571 |
| t [T1] | 4.34 | -3.30 – 11.98 | 0.264 |
| **t [T2]** | **11.82** | **3.85 – 19.79** | **0.004** |
| **t [T3]** | **25.67** | **17.70 – 33.64** | **<0.001** |
| **t [T4]** | **23.72** | **15.75 – 31.69** | **<0.001** |
| apiary [B] | -1.18 | -10.39 – 8.04 | 0.801 |
| apiary [C] | -0.93 | -10.14 – 8.29 | 0.843 |
| apiary [D] | -1.06 | -10.28 – 8.16 | 0.820 |
| apiary [E] | -0.84 | -10.06 – 8.38 | 0.858 |
| apiary [F] | -0.86 | -10.08 – 8.36 | 0.854 |
| t [T1] * apiary [B] | 1.24 | -9.81 – 12.30 | 0.824 |
| t [T2] * apiary [B] | 4.90 | -10.28 – 20.09 | 0.524 |
| **t [T3] * apiary [B]** | **24.09** | **8.91 – 39.28** | **0.002** |
| **t [T4] * apiary [B]** | **24.35** | **9.17 – 39.54** | **0.002** |
| t [T1] * apiary [C] | -1.99 | -12.79 – 8.81 | 0.717 |
| **t [T2] * apiary [C]** | **18.78** | **6.21 – 31.35** | **0.004** |
| **t [T3] * apiary [C]** | **52.55** | **39.08 – 66.01** | **<0.001** |
| **t [T4] * apiary [C]** | **49.67** | **34.60 – 64.74** | **<0.001** |
| t [T1] * apiary [D] | -2.19 | -12.99 – 8.61 | 0.690 |
| t [T2] * apiary [D] | 12.82 | -0.67 – 26.32 | 0.062 |
| **t [T3] * apiary [D]** | **57.31** | **42.19 – 72.42** | **<0.001** |
| **t [T4] * apiary [D]** | **70.11** | **55.00 – 85.23** | **<0.001** |
| t [T1] * apiary [E] | -1.69 | -12.49 – 9.11 | 0.758 |
| t [T2] * apiary [E] | 8.13 | -2.90 – 19.17 | 0.147 |
| **t [T3] * apiary [E]** | **21.73** | **10.69 – 32.77** | **<0.001** |
| **t [T4] * apiary [E]** | **16.53** | **4.99 – 28.07** | **0.005** |
| t [T1] * apiary [F] | -2.31 | -13.11 – 8.49 | 0.673 |
| t [T2] * apiary [F] | 9.15 | -2.42 – 20.72 | 0.120 |
| **t [T3] * apiary [F]** | **20.49** | **8.52 – 32.45** | **0.001** |
| **t [T4] * apiary [F]** | **19.46** | **7.50 – 31.43** | **0.002** |
| Random Effects | |  |  |
| σ2 | 59.73 |  |  |
| τ00 hive | 27.25 |  |  |
| ICC | 0.31 |  |  |
| N hive | 48 |  |  |
| Observations | 176 |  |  |
| Marginal R2 / Conditional R2 | 0.849 / 0.896 | |  |

Table S6: Details for the models used to analyze measurements of frame contents in July, using treatment and region as fixed factors. Significant effects (p < 0.05) indicated with bold.

| Model | Predictors | Estimates | CI | p |
| --- | --- | --- | --- | --- |
| Brood | **(Intercept)** | **109.11** | **80.65 – 137.57** | **<0.001** |
| R2=0.317 | Treatment [priming] | 3.80 | -24.00 – 31.60 | 0.780 |
| R2 adj=0.227 | Region [CD] | -39.33 | -79.56 – 0.90 | 0.055 |
|  | Region [EF] | 19.34 | -11.76 – 50.44 | 0.211 |
| Honey | **(Intercept)** | **212.36** | **84.87 – 339.86** | **0.002** |
| R2=0.522 | Treatment [priming] | 60.44 | -64.10 – 184.99 | 0.326 |
| R2 adj=0.46 | **Region [CD]** | **434.36** | **254.13 – 614.59** | **<0.001** |
|  | **Region [EF]** | **140.28** | **0.96 – 279.60** | **0.049** |
| Pollen | (Intercept) | 15.57 | -0.10 – 31.23 | 0.051 |
| R2=0.411 | Treatment [priming] | 10.88 | -4.42 – 26.18 | 0.155 |
| R2 adj=0.334 | **Region [CD]** | **40.88** | **18.73 – 63.03** | **0.001** |
|  | **Region [EF]** | **18.50** | **1.38 – 35.62** | **0.035** |
| Drones | (Intercept) | 0.51 | -2.24 – 3.25 | 0.705 |
| R2=0.329 | Treatment [priming] | -0.91 | -3.60 – 1.77 | 0.488 |
| R2 adj=0.242 | **Region [CD]** | **5.96** | **2.07 – 9.84** | **0.004** |
|  | Region [EF] | 1.75 | -1.25 – 4.76 | 0.239 |

Table S7: Model details for total honey yield, using treatment and region as fixed factors. Significant effects (p < 0.05) indicated with bold.

| Predictors | Estimates | CI | p |
| --- | --- | --- | --- |
| **(Intercept)** | **10.63** | **2.19 – 19.08** | **0.016** |
| Treatment [priming] | 2.52 | -6.18 – 11.22 | 0.552 |
| **Region [CD]** | **32.86** | **20.10 – 45.62** | **<0.001** |
| Region [EF] | 3.06 | -6.27 – 12.40 | 0.501 |
| R2 / R2 adjusted | 0.615 / 0.557 | |  |

## Details of pathogen models

Table S8: Model details for AFB binomial model, using treatment and worker stock source location as fixed factors and hive as random. Significant effects (p < 0.05) indicated with bold.

| Predictors | Odds Ratios | CI | p |
| --- | --- | --- | --- |
| **(Intercept)** | **0.06** | **0.01 – 0.23** | **<0.001** |
| Treatment [priming] | 1.53 | 0.46 – 5.13 | 0.493 |
| stock_loc [Site 3] | 2.80 | 0.49 – 15.88 | 0.245 |
| **stock_loc [Site 2]** | **14.09** | **2.96 – 67.01** | **0.001** |
| **T [T1]** | **0.03** | **0.01 – 0.12** | **<0.001** |
| **T [T2]** | **0.06** | **0.02 – 0.23** | **<0.001** |
| Random Effects |  |  |  |
| σ2 |  | 3.29 |  |
| τ00 hive |  | 2.01 |  |
| ICC |  | 0.38 |  |
| N hive |  | 48 |  |
| Observations |  | 373 |  |
| Marginal R2 / Conditional R2 | | 0.424 / 0.643 |  |

Table S9: Model details for Chalkbrood binomial model, using treatment, timepoint and region as fixed factors, hive as random factor. Significant effects (p < 0.05) indicated with bold.

| Predictors | Odds Ratios | CI | p |
| --- | --- | --- | --- |
| **(Intercept)** | **0.10** | **0.04 – 0.25** | **<0.001** |
| treatment [priming] | 1.46 | 0.70 – 3.03 | 0.314 |
| t [T1] | 0.89 | 0.42 – 1.87 | 0.753 |
| t [T2] | 1.29 | 0.58 – 2.89 | 0.532 |
| t [T3] | 0.63 | 0.24 – 1.66 | 0.348 |
| **t [T4]** | **2.44** | **1.10 – 5.41** | **0.029** |
| region [C-D] | 0.62 | 0.24 – 1.62 | 0.327 |
| region [E-F] | 1.12 | 0.48 – 2.60 | 0.797 |
| Random Effects |  |  |  |
| σ2 |  | 3.29 |  |
| τ00 hive |  | 0.64 |  |
| ICC |  | 0.16 |  |
| N hive |  | 48 |  |
| Observations |  | 526 |  |
| Marginal R2 / Conditional R2 | | 0.063 / 0.214 |  |

Table S10: Model details for BQCV mixed effects model using treatment, timepoint, apiary, and apiary-timepoint interaction as fixed effects, hive as random effect. Significant effects (p < 0.05) indicated with bold.

| Predictors | Estimates | CI | p |
| --- | --- | --- | --- |
| **(Intercept)** | **8.72** | **7.57 – 9.88** | **<0.001** |
| Treatment [priming] | 0.16 | -0.34 – 0.66 | 0.533 |
| t [T1] | 0.04 | -1.55 – 1.63 | 0.961 |
| t [T2] | -0.84 | -2.48 – 0.80 | 0.313 |
| **t [T3]** | **-4.17** | **-5.81 – -2.53** | **<0.001** |
| **t [T4]** | **-5.20** | **-6.85 – -3.56** | **<0.001** |
| apiary [B] | -0.40 | -2.00 – 1.19 | 0.618 |
| apiary [C] | -1.08 | -2.68 – 0.52 | 0.183 |
| apiary [D] | -1.01 | -2.61 – 0.59 | 0.215 |
| apiary [E] | -1.52 | -3.12 – 0.07 | 0.062 |
| apiary [F] | -0.67 | -2.27 – 0.93 | 0.411 |
| t [T1] * apiary [B] | -0.66 | -2.95 – 1.62 | 0.568 |
| t [T2] * apiary [B] | -1.63 | -4.64 – 1.38 | 0.286 |
| t [T3] * apiary [B] | 2.21 | -0.80 – 5.21 | 0.149 |
| t [T4] * apiary [B] | 2.15 | -0.85 – 5.16 | 0.159 |
| t [T1] * apiary [C] | -0.78 | -3.02 – 1.47 | 0.496 |
| t [T2] * apiary [C] | -2.35 | -4.90 – 0.19 | 0.070 |
| t [T3] * apiary [C] | -0.87 | -3.58 – 1.85 | 0.529 |
| t [T4] * apiary [C] | -2.14 | -5.16 – 0.88 | 0.164 |
| t [T1] * apiary [D] | 0.10 | -2.14 – 2.35 | 0.928 |
| **t [T2] * apiary [D]** | **-3.16** | **-5.87 – -0.45** | **0.023** |
| t [T3] * apiary [D] | 0.14 | -2.87 – 3.15 | 0.928 |
| t [T4] * apiary [D] | -1.12 | -4.12 – 1.89 | 0.464 |
| **t [T1] * apiary [E]** | **2.33** | **0.09 – 4.57** | **0.042** |
| **t [T2] * apiary [E]** | **2.60** | **0.32 – 4.88** | **0.026** |
| **t [T3] * apiary [E]** | **3.07** | **0.79 – 5.36** | **0.009** |
| **t [T4] * apiary [E]** | **4.35** | **1.98 – 6.73** | **<0.001** |
| t [T1] * apiary [F] | 0.63 | -1.61 – 2.88 | 0.578 |
| t [T2] * apiary [F] | 2.14 | -0.24 – 4.52 | 0.077 |
| **t [T3] * apiary [F]** | **2.49** | **0.04 – 4.93** | **0.046** |
| t [T4] * apiary [F] | 1.81 | -0.64 – 4.25 | 0.147 |
| Random Effects |  |  |  |
| σ2 |  | 2.61 |  |
| τ00 hive |  | 0.02 |  |
| ICC |  | 0.01 |  |
| N hive |  | 48 |  |
| Observations |  | 177 |  |
| Marginal R2 / Conditional R2 |  | 0.617 / 0.620 |  |

Table S11: Model details for DWV mixed effects model using treatment, timepoint, apiary, and apiary-timepoint interaction as fixed effects, hive as random effect. Significant effects (p < 0.05) indicated with bold.

| Predictors | Estimates | CI | p |
| --- | --- | --- | --- |
| **(Intercept)** | **10.59** | **8.43 – 12.76** | **<0.001** |
| Treatment [priming] | 0.90 | -0.18 – 1.97 | 0.101 |
| **t [T1]** | **-7.72** | **-10.48 – -4.96** | **<0.001** |
| **t [T2]** | **-9.35** | **-12.22 – -6.48** | **<0.001** |
| **t [T3]** | **-10.63** | **-13.49 – -7.76** | **<0.001** |
| **t [T4]** | **-6.25** | **-9.12 – -3.38** | **<0.001** |
| apiary [B] | -1.77 | -4.74 – 1.20 | 0.240 |
| **apiary [C]** | **-3.50** | **-6.47 – -0.53** | **0.021** |
| **apiary [D]** | **-4.21** | **-7.18 – -1.24** | **0.006** |
| apiary [E] | -2.68 | -5.65 – 0.29 | 0.076 |
| **apiary [F]** | **-3.65** | **-6.62 – -0.68** | **0.016** |
| t [T1] * apiary [B] | 0.51 | -3.47 – 4.50 | 0.799 |
| t [T2] * apiary [B] | 0.80 | -4.55 – 6.15 | 0.768 |
| t [T3] * apiary [B] | 3.11 | -2.24 – 8.47 | 0.252 |
| t [T4] * apiary [B] | 2.74 | -2.61 – 8.09 | 0.313 |
| **t [T1] * apiary [C]** | **7.37** | **3.47 – 11.28** | **<0.001** |
| **t [T2] * apiary [C]** | **8.75** | **4.26 – 13.23** | **<0.001** |
| **t [T3] * apiary [C]** | **8.56** | **3.77 – 13.35** | **0.001** |
| **t [T4] * apiary [C]** | **11.41** | **6.06 – 16.77** | **<0.001** |
| **t [T1] * apiary [D]** | **7.97** | **4.07 – 11.88** | **<0.001** |
| t [T2] * apiary [D] | 2.94 | -1.85 – 7.74 | 0.227 |
| **t [T3] * apiary [D]** | **5.47** | **0.12 – 10.82** | **0.045** |
| t [T4] * apiary [D] | 2.96 | -2.39 – 8.30 | 0.276 |
| t [T1] * apiary [E] | 1.09 | -2.81 – 5.00 | 0.582 |
| **t [T2] * apiary [E]** | **4.33** | **0.35 – 8.31** | **0.033** |
| **t [T3] * apiary [E]** | **12.07** | **8.09 – 16.05** | **<0.001** |
| **t [T4] * apiary [E]** | **5.52** | **1.36 – 9.67** | **0.010** |
| t [T1] * apiary [F] | 0.44 | -3.46 – 4.34 | 0.824 |
| **t [T2] * apiary [F]** | **9.78** | **5.62 – 13.94** | **<0.001** |
| **t [T3] * apiary [F]** | **18.99** | **14.70 – 23.28** | **<0.001** |
| **t [T4] * apiary [F]** | **13.44** | **9.15 – 17.73** | **<0.001** |
| Random Effects |  |  |  |
| σ2 |  | 7.80 |  |
| τ00 hive |  | 1.22 |  |
| ICC |  | 0.14 |  |
| N hive |  | 48 |  |
| Observations |  | 176 |  |
| Marginal R2 / Conditional R2 |  | 0.635 / 0.685 |  |

Table S12: Model details for SBV mixed effects model using treatment, timepoint, apiary, and apiary-timepoint interaction as fixed effects, hive as random effect. Significant effects (p < 0.05) indicated with bold.

| Predictors | Estimates | CI | p | |
| --- | --- | --- | --- | --- |
| (Intercept) | 6.08 | 3.13 – 9.03 | <0.001 |  |
| Treatment [priming] | -0.61 | -2.12 – 0.89 | 0.423 |  |
| **t [T1]** | **6.65** | **2.97 – 10.33** | **<0.001** |  |
| **t [T2]** | **6.22** | **2.39 – 10.05** | **0.002** |  |
| **t [T3]** | **5.09** | **1.26 – 8.92** | **0.010** |  |
| t [T4] | -0.90 | -4.72 – 2.93 | 0.644 |  |
| apiary [B] | -0.52 | -4.56 – 3.52 | 0.800 |  |
| **apiary [C]** | **4.09** | **0.05 – 8.13** | **0.047** |  |
| apiary [D] | 0.89 | -3.15 – 4.93 | 0.664 |  |
| apiary [E] | -0.85 | -4.88 – 3.19 | 0.679 |  |
| **apiary [F]** | **4.34** | **0.30 – 8.38** | **0.035** |  |
| t [T1] * apiary [B] | -0.48 | -5.79 – 4.84 | 0.859 |  |
| t [T2] * apiary [B] | -3.08 | -10.25 – 4.10 | 0.398 |  |
| t [T3] * apiary [B] | -0.67 | -7.84 – 6.51 | 0.855 |  |
| t [T4] * apiary [B] | 0.30 | -6.87 – 7.48 | 0.934 |  |
| **t [T1] * apiary [C]** | **-8.90** | **-14.11 – -3.70** | **0.001** |  |
| **t [T2] * apiary [C]** | **-9.17** | **-15.16 – -3.17** | **0.003** |  |
| **t [T3] * apiary [C]** | **-8.39** | **-14.80 – -1.98** | **0.011** |  |
| t [T4] * apiary [C] | -6.09 | -13.26 – 1.07 | 0.095 |  |
| t [T1] * apiary [D] | -4.00 | -9.21 – 1.20 | 0.131 |  |
| **t [T2] * apiary [D]** | **-8.95** | **-15.37 – -2.54** | **0.007** |  |
| t [T3] * apiary [D] | -2.64 | -9.81 – 4.52 | 0.467 |  |
| t [T4] * apiary [D] | -3.54 | -10.70 – 3.62 | 0.330 |  |
| t [T1] * apiary [E] | 3.24 | -1.96 – 8.45 | 0.220 |  |
| t [T2] * apiary [E] | -0.65 | -5.96 – 4.66 | 0.809 |  |
| t [T3] * apiary [E] | 2.65 | -2.66 – 7.96 | 0.326 |  |
| t [T4] * apiary [E] | 3.22 | -2.32 – 8.76 | 0.252 |  |
| t [T1] * apiary [F] | -5.08 | -10.29 – 0.12 | 0.055 |  |
| **t [T2] * apiary [F]** | **-10.52** | **-16.07 – -4.97** | **<0.001** |  |
| t [T3] * apiary [F] | -4.29 | -10.02 – 1.43 | 0.141 |  |
| t [T4] * apiary [F] | -1.73 | -7.46 – 4.00 | 0.551 |  |
| Random Effects |  |  |  |  |
| σ2 | 13.87 |  |  |  |
| τ00 hive | 2.82 |  |  |  |
| ICC | 0.17 |  |  |  |
| N hive | 48 |  |  |  |
| Observations | 176 |  |  |  |
| Marginal R2 / Conditional R2 | 0.358 / 0.467 | |  |  |

Table S13: Model details for *Nosema apis* mixed effects model using treatment, timepoint, apiary, and apiary-timepoint interaction as fixed effects, hive as random effect. Significant effects (p < 0.05) indicated with bold.

| Predictors | Estimates | CI | p |
| --- | --- | --- | --- |
| **(Intercept)** | **5.79** | **4.27 – 7.30** | **<0.001** |
| Treatment [priming] | 0.25 | -0.48 – 0.97 | 0.498 |
| **t [T1]** | **-2.82** | **-4.80 – -0.84** | **0.005** |
| t [T2] | -0.48 | -2.53 – 1.58 | 0.648 |
| **t [T3]** | **-2.95** | **-5.00 – -0.90** | **0.005** |
| **t [T4]** | **-3.44** | **-5.49 – -1.39** | **0.001** |
| apiary [B] | -1.07 | -3.15 – 1.01 | 0.312 |
| apiary [C] | -0.31 | -2.40 – 1.77 | 0.765 |
| apiary [D] | 0.05 | -2.03 – 2.14 | 0.959 |
| **apiary [E]** | **-3.30** | **-5.38 – -1.22** | **0.002** |
| **apiary [F]** | **2.60** | **0.51 – 4.68** | **0.015** |
| **t [T1] * apiary [B]** | **3.84** | **0.99 – 6.69** | **0.009** |
| t [T2] * apiary [B] | 0.21 | -3.60 – 4.02 | 0.914 |
| **t [T3] * apiary [B]** | **5.75** | **1.94 – 9.56** | **0.003** |
| t [T4] * apiary [B] | 0.37 | -3.44 – 4.18 | 0.849 |
| t [T1] * apiary [C] | 1.05 | -1.74 – 3.84 | 0.459 |
| t [T2] * apiary [C] | -1.18 | -4.38 – 2.02 | 0.469 |
| **t [T3] * apiary [C]** | **5.39** | **1.97 – 8.80** | **0.002** |
| **t [T4] * apiary [C]** | **4.14** | **0.33 – 7.96** | **0.033** |
| t [T1] * apiary [D] | 2.06 | -0.74 – 4.85 | 0.147 |
| t [T2] * apiary [D] | 2.62 | -0.79 – 6.04 | 0.131 |
| t [T3] * apiary [D] | 2.59 | -1.22 – 6.39 | 0.181 |
| t [T4] * apiary [D] | 2.76 | -1.04 – 6.57 | 0.154 |
| **t [T1] * apiary [E]** | **6.26** | **3.47 – 9.06** | **<0.001** |
| **t [T2] * apiary [E]** | **4.34** | **1.49 – 7.19** | 0.003 |
| **t [T3] * apiary [E]** | **5.79** | **2.94 – 8.64** | **<0.001** |
| **t [T4] * apiary [E]** | **6.02** | **3.05 – 8.98** | **<0.001** |
| t [T1] * apiary [F] | -0.66 | -3.45 – 2.14 | 0.644 |
| **t [T2] * apiary [F]** | **-3.92** | **-6.89 – -0.95** | **0.010** |
| t [T3] * apiary [F] | 0.56 | -2.50 – 3.63 | 0.716 |
| t [T4] * apiary [F] | -2.55 | -5.61 – 0.51 | 0.102 |
| Random Effects |  |  |  |
| σ2 |  | 4.00 |  |
| τ00 hive |  | 0.44 |  |
| ICC |  | 0.10 |  |
| N hive |  | 48 |  |
| Observations |  | 176 |  |
| Marginal R2 / Conditional R2 |  | 0.368 / 0.431 |  |

Table S14: Model details for *Nosema ceranae* mixed effects model using treatment, timepoint, apiary, and apiary-timepoint interaction as fixed effects, hive as random effect. Significant effects (p < 0.05) indicated with bold.

| Predictors | Estimates | CI | p |
| --- | --- | --- | --- |
| (Intercept) | 0.91 | -1.90 – 3.72 | 0.525 |
| Treatment [priming] | 1.22 | -0.23 – 2.67 | 0.098 |
| **t [T1]** | **6.72** | **3.24 – 10.20** | **<0.001** |
| **t [T2]** | **22.31** | **18.69 – 25.94** | **<0.001** |
| **t [T3]** | **19.49** | **15.87 – 23.11** | **<0.001** |
| **t [T4]** | **14.42** | **10.80 – 18.04** | **<0.001** |
| apiary [B] | 2.87 | -0.97 – 6.71 | 0.142 |
| apiary [C] | -1.20 | -5.04 – 2.64 | 0.537 |
| apiary [D] | 0.68 | -3.17 – 4.52 | 0.729 |
| apiary [E] | -0.30 | -4.15 – 3.54 | 0.876 |
| apiary [F] | 0.24 | -3.60 – 4.08 | 0.903 |
| t [T1] * apiary [B] | -4.06 | -9.08 – 0.97 | 0.113 |
| t [T2] * apiary [B] | -5.85 | -12.65 – 0.94 | 0.091 |
| **t [T3] * apiary [B]** | **-9.06** | **-15.86 – -2.27** | **0.009** |
| **t [T4] * apiary [B]** | **-7.12** | **-13.91 – -0.32** | **0.040** |
| t [T1] * apiary [C] | -2.13 | -7.05 – 2.79 | 0.394 |
| **t [T2] * apiary [C]** | **-7.13** | **-12.81 – -1.46** | **0.014** |
| t [T3] * apiary [C] | -1.43 | -7.50 – 4.63 | 0.641 |
| t [T4] * apiary [C] | 6.60 | -0.19 – 13.38 | 0.057 |
| t [T1] * apiary [D] | -2.70 | -7.62 – 2.22 | 0.280 |
| **t [T2] * apiary [D]** | **-12.72** | **-18.79 – -6.65** | **<0.001** |
| **t [T3] * apiary [D]** | **-11.07** | **-17.85 – -4.29** | **0.002** |
| **t [T4] * apiary [D]** | **-8.60** | **-15.38 – -1.81** | **0.013** |
| t [T1] * apiary [E] | -0.50 | -5.42 – 4.42 | 0.841 |
| t [T2] * apiary [E] | 2.02 | -3.01 – 7.04 | 0.428 |
| t [T3] * apiary [E] | -0.61 | -5.63 – 4.42 | 0.812 |
| t [T4] * apiary [E] | 4.89 | -0.35 – 10.13 | 0.067 |
| **t [T1] * apiary [F]** | **9.16** | **4.24 – 14.08** | **<0.001** |
| t [T2] * apiary [F] | -3.21 | -8.46 – 2.03 | 0.228 |
| t [T3] * apiary [F] | -4.50 | -9.92 – 0.92 | 0.103 |
| t [T4] * apiary [F] | 3.83 | -1.59 – 9.25 | 0.165 |
| Random Effects |  |  |  |
| σ2 |  | 12.40 |  |
| τ00 hive |  | 2.70 |  |
| ICC |  | 0.18 |  |
| N hive |  | 48 |  |
| Observations |  | 176 |  |
| Marginal R2 / Conditional R2 |  | 0.819 / 0.851 |  |

## Details of gene expression models

Table S15: Model details for *Apidaecin* expression mixed effects model, using treatment, BQCV, timepoint and region as fixed effects, hive as random. Significant effects (p < 0.05) indicated with bold.

| Predictors | Estimates | CI | p |
| --- | --- | --- | --- |
| **(Intercept)** | **-12.50** | **-13.02 – -11.97** | **<0.001** |
| Treatment [priming] | 0.06 | -0.28 – 0.41 | 0.712 |
| **BQCV** | **-0.10** | **-0.18 – -0.01** | **0.029** |
| **Region [CD]** | **0.49** | **0.00 – 0.97** | **0.049** |
| Region [EF] | -0.11 | -0.60 – 0.38 | 0.664 |
| **time [T2]** | **1.05** | **0.57 – 1.53** | **<0.001** |
| Region [CD] * time [T2] | 0.09 | -0.65 – 0.83 | 0.807 |
| **Region [EF] * time [T2]** | **-0.82** | **-1.45 – -0.18** | **0.013** |
| Random Effects |  |  |  |
| σ2 | 0.29 |  |  |
| τ00 hive | 0.15 |  |  |
| ICC | 0.34 |  |  |
| N hive | 46 |  |  |
| Observations | 75 |  |  |
| Marginal R2 / Conditional R2 | 0.319 / 0.547 |  |  |

Table S16: Model details for *Hymenoptaecin* expression mixed effects model, using treatment, timepoint and region as fixed effects, hive as random. Significant effects (p < 0.05) indicated with bold.

| Predictors | Estimates | CI | p |
| --- | --- | --- | --- |
| **(Intercept)** | **-8.40** | **-9.24 – -7.56** | **<0.001** |
| Treatment [priming] | -0.26 | -0.96 – 0.44 | 0.460 |
| **T [T2]** | **2.66** | **1.39 – 3.93** | **<0.001** |
| **Region [CD]** | **-1.09** | **-2.17 – -0.01** | **0.048** |
| **Region [EF]** | **-1.12** | **-2.22 – -0.02** | **0.046** |
| T [T2] * Region [CD] | -0.01 | -1.87 – 1.86 | 0.995 |
| **T [T2] * Region [EF]** | **-1.95** | **-3.66 – -0.25** | **0.026** |
| Random Effects |  |  |  |
| σ2 | 2.27 |  |  |
| τ00 Hive | 0.00 |  |  |
| N Hive | 46 |  |  |
| Observations | 75 |  |  |
| Marginal R2 / Conditional R2 | 0.419 / NA |  |  |

Table S17: Model details for *PEPCK* expression mixed effects model, using treatment, timepoint and region as fixed effects, hive as random. Significant effects (p < 0.05) indicated with bold.

| Predictors | Estimates | CI | p |
| --- | --- | --- | --- |
| **(Intercept)** | **-7.42** | **-7.91 – -6.92** | **<0.001** |
| Treatment [priming] | 0.14 | -0.33 – 0.60 | 0.557 |
| **T [T2]** | **1.10** | **0.55 – 1.64** | **<0.001** |
| **Region [CD]** | **-0.89** | **-1.51 – -0.27** | **0.005** |
| **Region [EF]** | **-0.68** | **-1.31 – -0.05** | **0.034** |
| T [T2] * Region [CD] | -0.72 | -1.54 – 0.09 | 0.080 |
| T [T2] * Region [EF] | -0.29 | -1.02 – 0.43 | 0.417 |
| Random Effects |  |  |  |
| σ2 | 0.37 |  |  |
| τ00 Hive | 0.37 |  |  |
| ICC | 0.50 |  |  |
| N Hive | 46 |  |  |
| Observations | 75 |  |  |
| Marginal R2 / Conditional R2 | 0.348 / 0.672 |  |  |

Table S18: Model details for *PER* expression mixed effects model, using treatment, timepoint and region as fixed effects, hive as random. Significant effects (p < 0.05) indicated with bold text.

| Predictors | Estimates | CI | p |
| --- | --- | --- | --- |
| **(Intercept)** | **-8.85** | **-9.78 – -7.91** | **<0.001** |
| Treatment [priming] | -0.10 | -0.88 – 0.68 | 0.801 |
| T [T2] | 0.45 | -0.97 – 1.86 | 0.530 |
| Region [CD] | -0.69 | -1.89 – 0.52 | 0.260 |
| Region [EF] | 0.02 | -1.20 – 1.25 | 0.970 |
| T [T2] * Region [CD] | 0.70 | -1.37 – 2.78 | 0.502 |
| T [T2] * Region [EF] | -0.16 | -2.06 – 1.75 | 0.871 |
| Random Effects |  |  |  |
| σ2 | 2.82 |  |  |
| τ00 Hive | 0.00 |  |  |
| N Hive | 46 |  |  |
| Observations | 75 |  |  |
| Marginal R2 / Conditional R2 | 0.055 / NA |  |  |

Table S19: Model details for *PGRP-LC* expression mixed effects model, using treatment, BQCV, timepoint and region as fixed effects, hive as random. Significant effects (p < 0.05) indicated with bold.

| Predictors | Estimates | CI | p |
| --- | --- | --- | --- |
| **(Intercept)** | **-7.73** | **-7.92 – -7.55** | **<0.001** |
| Treatment [priming] | 0.03 | -0.13 – 0.18 | 0.735 |
| **BQCV** | **0.03** | **0.00 – 0.05** | **0.024** |
| **Region [CD]** | **-0.20** | **-0.40 – -0.00** | **0.048** |
| Region [EF] | 0.14 | -0.06 – 0.34 | 0.171 |
| **time [T2]** | **0.16** | **0.05 – 0.27** | **0.006** |
| **Region [CD] * time [T2]** | **-0.48** | **-0.65 – -0.30** | **<0.001** |
| Region [EF] * time [T2] | -0.08 | -0.23 – 0.06 | 0.256 |
| Random Effects |  |  |  |
| σ2 | 0.01 |  |  |
| τ00 hive | 0.06 |  |  |
| ICC | 0.81 |  |  |
| N hive | 46 |  |  |
| Observations | 75 |  |  |
| Marginal R2 / Conditional R2 | 0.335 / 0.876 |  |  |

Table S20: Model details for *PPO* expression mixed effects model, using treatment, timepoint and region as fixed effects, hive as random. Significant effects (p < 0.05) indicated with bold.

| Predictors | Estimates | CI | p |
| --- | --- | --- | --- |
| **(Intercept)** | **-4.90** | **-5.25 – -4.55** | **<0.001** |
| Treatment [priming] | 0.12 | -0.18 – 0.42 | 0.434 |
| **T [T2]** | **-1.03** | **-1.52 – -0.54** | **<0.001** |
| **Region [CD]** | **-1.41** | **-1.85 – -0.96** | **<0.001** |
| **Region [EF]** | **-0.74** | **-1.19 – -0.28** | **0.002** |
| **T [T2] * Region [CD]** | **0.88** | **0.16 – 1.60** | **0.017** |
| **T [T2] * Region [EF]** | **0.75** | **0.09 – 1.40** | **0.026** |
| Random Effects |  |  |  |
| σ2 | 0.32 |  |  |
| τ00 Hive | 0.06 |  |  |
| ICC | 0.16 |  |  |
| N Hive | 46 |  |  |
| Observations | 75 |  |  |
| Marginal R2 / Conditional R2 | 0.413 / 0.508 |  |  |

Table S21: Model details for *trynity* expression mixed effects model, using treatment, timepoint and region as fixed effects, hive as random. Significant effects (p < 0.05) indicated with bold text.

| Predictors | Estimates | CI | p |
| --- | --- | --- | --- |
| **(Intercept)** | **-5.96** | **-6.53 – -5.39** | **<0.001** |
| Treatment [priming] | 0.00 | -0.47 – 0.48 | 0.989 |
| **T [T2]** | **1.58** | **0.73 – 2.44** | **<0.001** |
| Region [CD] | 0.12 | -0.61 – 0.85 | 0.748 |
| Region [EF] | 0.67 | -0.08 – 1.41 | 0.078 |
| T [T2] * Region [CD] | -0.60 | -1.86 – 0.65 | 0.339 |
| T [T2] * Region [EF] | -1.02 | -2.17 – 0.13 | 0.081 |
| Random Effects |  |  |  |
| σ2 | 1.03 |  |  |
| τ00 Hive | 0.01 |  |  |
| ICC | 0.01 |  |  |
| N Hive | 46 |  |  |
| Observations | 75 |  |  |
| Marginal R2 / Conditional R2 | 0.242 / 0.249 |  |  |

# References

Blanchard, P., Ribière, M., Celle, O., Lallemand, P., Schurr, F., Olivier, V., et al. (2007). Evaluation of a real-time two-step RT-PCR assay for quantitation of Chronic bee paralysis virus (CBPV) genome in experimentally-infected bee tissues and in life stages of a symptomatic colony. *J Virol Methods* 141, 7–13. doi: 10.1016/J.JVIROMET.2006.11.021.

Chantawannakul, P., Ward, L., Boonham, N., and Brown, M. (2006). A scientific note on the detection of honeybee viruses using real-time PCR (TaqMan) in Varroa mites collected from a Thai honeybee (Apis mellifera) apiary. *J Invertebr Pathol* 91, 69–73. doi: 10.1016/J.JIP.2005.11.001.

Chen, Y. P., Higgins, J. A., and Feldlaufer, M. F. (2005). Quantitative Real-Time Reverse Transcription-PCR Analysis of Deformed Wing Virus Infection in the Honeybee (Apis mellifera L.). *Appl Environ Microbiol* 71, 436. doi: 10.1128/AEM.71.1.436-441.2005.

Cornman, R. S., Lopez, D., and Evans, J. D. (2013). Transcriptional response of honey bee larvae infected with the bacterial pathogen *Paenibacillus larvae*. *PLoS One* 8. doi: 10.1371/JOURNAL.PONE.0065424.

D’Alvise, P., Seeburger, V., Gihring, K., Kieboom, M., and Hasselmann, M. (2019). Seasonal dynamics and co-occurrence patterns of honey bee pathogens revealed by high-throughput RT-qPCR analysis. *Ecol Evol* 9, 10241–10252. doi: 10.1002/ece3.5544.

Evans, J. D., Aronstein, K., Chen, Y. P., Hetru, C., Imler, J. L., Jiang, H., et al. (2006). Immune pathways and defence mechanisms in honey bees *Apis mellifera*. *Insect Mol Biol* 15, 645–656. doi: 10.1111/j.1365-2583.2006.00682.x.

Huang, W. F., and Solter, L. F. (2013). Comparative development and tissue tropism of Nosema apis and Nosema ceranae. *J Invertebr Pathol* 113, 35–41. doi: 10.1016/J.JIP.2013.01.001.

Jennette, M. R. (2017). High Fructose Corn Syrup Down-Regulates the Glycolysis Pathway in Apis mellifera. Available at: https://vc.bridgew.edu/honors_proj/225.

Jeon, J. H., Moon, K. H., Kim, Y. H., and Kim, Y. H. (2020). Reference gene selection for qRT-PCR analysis of season- and tissue-specific gene expression profiles in the honey bee Apis mellifera. *Scientific Reports 2020 10:1* 10, 1–13. doi: 10.1038/s41598-020-70965-4.

Mcmahon, D. P., Fürst, M. A., Caspar, J., Theodorou, P., Brown, M. J. F., and Paxton, R. J. (2015). A sting in the spit: widespread cross-infection of multiple RNA viruses across wild and managed bees. *Journal of Animal Ecology* 84, 615–624. doi: 10.1111/1365-2656.12345.

Roetschi, A., Berthoud, H., Kuhn, R., and Imdorf, A. (2008). Infection rate based on quantitative real-time PCR of Melissococcus plutonius, the causal agent of European foulbrood, in honeybee colonies before and after apiary sanitation. *Apidologie* 39, 362–371. doi: 10.1051/APIDO:200819.

Rossi, F., Amadoro, C., Ruberto, A., and Ricchiuti, L. (2018). Evaluation of quantitative PCR (qPCR) Paenibacillus larvae targeted assays and definition of optimal conditions for its detection/quantification in honey and hive debris. *Insects* 9. doi: 10.3390/insects9040165.

Siebert, S. C., Kanga, L. H. B., Basha, S. M., and Legaspi, J. C. (2020). Molecular Assessment of Genes Linked to Immune Response Traits of Honey Bees in Conventional and Organically Managed Apiaries. *Insects* 11, 1–12. doi: 10.3390/INSECTS11090637.

VanEngelsdorp, D., Evans, J. D., Saegerman, C., Mullin, C., Haubruge, E., Nguyen, B. K., et al. (2009). Colony Collapse Disorder: A Descriptive Study. *PLoS One* 4, e6481. doi: 10.1371/journal.pone.0006481.
